# Atypical Clinical Manifestations of Loiasis and Their Relevance for Endemic Populations

**DOI:** 10.1093/ofid/ofz417

**Published:** 2019-11-01

**Authors:** Kevin G Buell, Charles Whittaker, Cédric B Chesnais, Paul D Jewell, Sébastien D S Pion, Martin Walker, Maria-Gloria Basáñez, Michel Boussinesq

**Affiliations:** 1 Department of Infectious Disease Epidemiology, London Centre for Neglected Tropical Disease Research and MRC Centre for Global Infectious Disease Analysis, Faculty of Medicine (St Mary’s Campus), Imperial College London, London, UK; 2 Institut de Recherche pour le Développement (IRD), UMI 233-INSERM U1175-Montpellier University, Montpellier, France; 3 Department of Pathobiology and Population Sciences, London Centre for Neglected Tropical Disease Research, Royal Veterinary College, Hatfield, UK

**Keywords:** atypical clinical manifestations, case reports, *Loa loa*, microfilaremia, systematic review

## Abstract

**Background:**

Loiasis is mostly considered a relatively benign infection when compared with other filarial and parasitic diseases, with Calabar swellings and eyeworm being the most common signs. Yet, there are numerous reports in the literature of more serious sequelae. Establishing the relationship between infection and disease is a crucial first step toward estimating the burden of loiasis.

**Methods:**

We conducted a systematic review of case reports containing 329 individuals and detailing clinical manifestations of loiasis with a focus on nonclassical, atypical presentations.

**Results:**

Results indicate a high proportion (47%) of atypical presentations in the case reports identified, encompassing a wide range of cardiac, respiratory, gastrointestinal, renal, neurological, ophthalmological, and dermatological pathologies. Individuals with high microfilarial densities and residing in an endemic country were at greater risk of suffering from atypical manifestations.

**Conclusions:**

Our findings have important implications for understanding the clinical spectrum of conditions associated with *Loa loa* infection, which extends well beyond the classical eyeworm and Calabar swellings. As case reports may overestimate the true rate of atypical manifestations in endemic populations, large-scale, longitudinal clinico-epidemiological studies will be required to refine our estimates and demonstrate causality between loiasis and the breadth of clinical manifestations reported. Even if the rates of atypical presentations were found to be lower, given that residents of loiasis-endemic areas are both numerous and the group most at risk of severe atypical manifestations, our conclusions support the recognition of loiasis as a significant public health burden across Central Africa.

## Teaser

Our (largest-to-date) systematic review on loiasis case reports assessed atypical clinical manifestations, that is, those not simply involving Calabar swellings or eyeworm passage. Forty-seven percent (154/329) of patients presented atypically, mostly involving vital organs. High microfilaremia was identified as a risk factor.

The filarial nematode *Loa loa*, transmitted between humans by *Chrysops* (tabanid) flies, causes loiasis, a disease endemic to forested areas of Central Africa [1]. An estimated 14 million people currently reside in high-risk areas (where *L. loa* microfilaremia prevalence is ≥20%), for example, in Cameroon, Gabon, and Democratic Republic of the Congo [2]. Loiasis is known for 2 hallmark signs, namely “Calabar” swellings (localized transient subcutaneous swellings) and adult worm migration under the bulbar conjunctiva (“eyeworm”). Despite being highly prevalent across parts of Central Africa, and estimated as the third most common reason for medical consultation in heavily affected areas, loiasis is perceived as a relatively benign condition [3]. In contrast to other filariases such as onchocerciasis and lymphatic filariasis, it remains absent from the World Health Organization’s list of prioritized neglected tropical diseases [4]. A recently demonstrated association between heavy microfilarial carriage and increased human mortality [5] has led to calls for recognition of loiasis as a significant public health problem [6].

Research on loiasis has been primarily concerned with the impediment it poses to mass treatment with ivermectin for the control of onchocerciasis in Central Africa [7]; individuals with high levels of circulating microfilariae (mf; the adult worm’s progeny) in the blood have an increased risk of developing severe adverse events (SAEs) after microfilaricidal treatment [8, 9]. Research on clinical manifestations has focused on Calabar swellings and eyeworm migration, including work from endemic areas [10, 11] and retrospective syntheses of cases presenting to clinical consultation in nonendemic countries [12–18]. However, the full spectrum of clinical manifestations remains poorly defined, with an array of both benign and severe cardiac, respiratory, renal, gastrointestinal, ophthalmic, neurological, and other manifestations having been observed in individual case reports.

Aiming to characterize the full spectrum of disease associated with loiasis and explore its determinants, we undertook a systematic review and analysis of published case reports containing individual patient data (IPD) on clinical manifestations. We present an overview of the atypical disease manifestations of loiasis (defined as presentations featuring signs other than Calabar swellings and/or eyeworm) and assess the influence of different factors on the propensity of individuals to present typically (Calabar swellings and/or eyeworm) or atypically (all other manifestations attributed to loiasis by the reviewed papers’ authors).

## METHODS

### Systematic Review

We conducted a systematic review of published case reports and case series containing IPD on loiasis, without any restrictions on publication dates. The search, carried out in Embase, Medline (PubMed), Global Health, Web of Science, and Scopus on December 22, 2017, used the following medical subject headings: “*Loa loa*” or “loiasis” or “loaiasis” or “filaria* loa” or “*loa* filaria*” or “eyeworm*” or “eye worm*” or “calabar swelling*.”

Three of the 5 databases could be searched using predefined subject headings. In Embase and Global Health, “*Loa*,” “*Loa loa*,” and “loiasis” were used. For Medline (Pubmed), “*Loa*” and “loiasis” were selected. For these, subject headings were combined with medical subject headings using the “OR” function.

**Figure 1.**
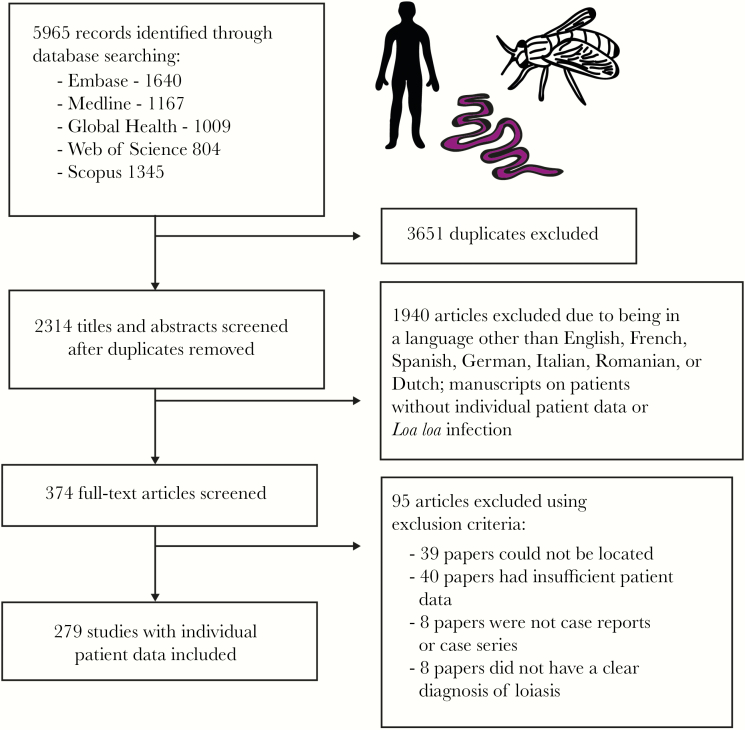
Systematic review methodology. Flow diagram showing the number of papers identified, screened, assessed for eligibility, and included in the systematic review of individual patient data from loiasis case reports.

The search yielded 5965 articles; 3651 duplicates were removed. Case reports and case series containing IPD written in English, French, Spanish, German, Italian, Romanian, and Dutch were independently reviewed for eligibility by 2 authors. Articles were excluded if the patient did not show evidence of *Loa loa* infection (defined as the presence/history of subconjunctival or subcutaneous adult worm migration, Calabar swellings, microfilaremia, or parasite identification following worm extraction, if undertaken) or when the manuscript contained insufficient IPD metadata (eg, no information on cause of consultation, clinical presentation, or diagnostic methods used). Disagreements were resolved by discussion and, if required, arbitration by a third author. A total of 279 articles were retained for data extraction and analysis (Figure 1). The full list of included papers is given in the Supplementary Data (“Literature Review”). The protocol was registered prospectively with PROSPERO [International Prospective Register of Systematic Reviews] (March 4, 2018, CRD42018092232) and followed the guidelines set out by PRISMA [Preferred Reporting Items for Systematic Reviews and Meta-Analyses] for systematic reviews of IPD.

### Patient Characteristics

Three authors independently extracted de-identified IPD: patient demographics (age, sex, residency status), physical examination, manifestations attributed to loiasis, blood tests (including eosinophilia and assessment of microfilaremia), diagnostic imaging, histopathology, and treatment. Antifilarial treatment offered to patients included diethylcarbamazine (DEC; most frequently), ivermectin, albendazole, mebendazole, and imatinib. Where particular data were not available for a given patient, manifestations not reported by the paper’s author(s) were considered to be absent. Co-infections were also recorded.

The residency status of patients was assigned to 3 categories: locals residing in an endemic country (born and living in an endemic region), locals residing in a nonendemic country (born in an endemic region but living in a nonendemic region at the time of consultation), and expatriates (born in a nonendemic region but having spent a period of time in an endemic region). Manifestations were categorized into typical (Calabar swelling and eyeworm) and atypical (all other manifestations attributed to loiasis, including nonclassical ocular signs). When microfilarial densities were available, individuals were assigned to 1 of the following 3 categories: zero (amicrofilaremic individuals, with no detectable mf), low (>0 but <8000 mf/mL blood), or high (≥8000 mf/mL blood). The threshold of 8000 mf/mL was chosen, as individuals with higher densities have an increased risk of marked adverse events (ie, functional impairment for several days) after ivermectin treatment [8]. Eosinophilia was defined as a peripheral eosinophil blood count >0.5×10^9^ cells or a peripheral blood eosinophil count ≥6% [19].

### Data Analysis

Descriptive statistical analyses (% of cases with each condition by organ system) were undertaken to characterize the full spectrum of clinical conditions associated with *L. loa* infection. Pearson’s chi-square test was used to assess whether the proportion of patients presenting atypically varied across the different microfilarial density categories. Multivariable logistic regression models were used to investigate the existence of associations between demographic factors (age, sex, residency status), microfilarial density, and type of presentation (atypical or typical), yielding odds ratio (OR) values and their associated 95% confidence intervals (CIs). The statistical methodology is described in the Supplementary Data (“Statistical Analyses”). All analyses were conducted using R, version 3.4.4 (https://www.r-project.org/).

## RESULTS

### Patient Demographics

Data from 329 patients were obtained. A total of 169 patients (51.4%) were classified as locals, 156 (47.4%) as expatriates, and 4 (1.2%) could not be assigned due to missing information. From the local patients, 61 (36.1%) were living in an endemic country, 99 (58.6%) were not living in an endemic country at the time of presenting, and 9 (5.3%) could not be classified due to missing information. A total of 148 patients (45.0%) were female, 170 (51.7%) were male, and there were 11 (3.3%) instances in which the patient’s sex was not stated. The patient’s age was reported in 302 (91.8%) cases, with a median (interquartile range [IQR]) of 32 (24–41) years.

### Clinical Manifestations

**Figure 2.**
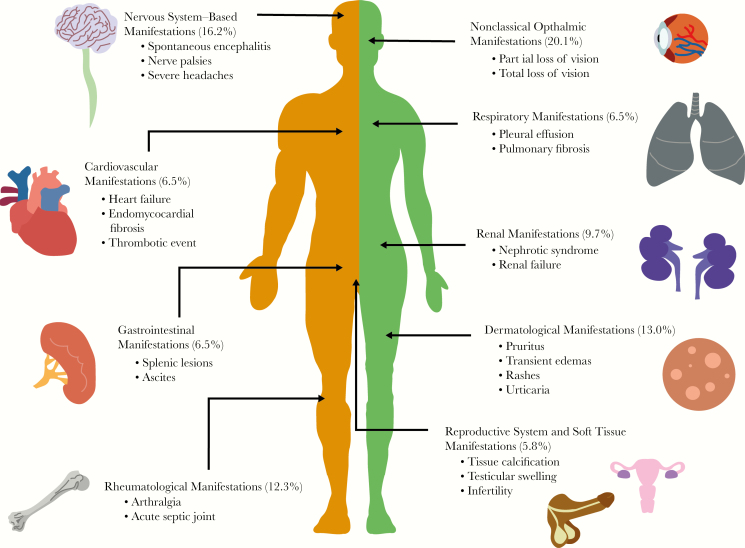
The spectrum of atypical clinical manifestations of loiasis. Schematic visualization of some of the atypical presentations of loiasis identified through the systematic literature review, highlighting the key vital organs and systems involved (confirmed by presence of adult *Loa loa* and/or microfilarial stages), the percentage of patients presenting with manifestations pertaining to that system, and some of the most common atypical presentations observed for that system.

A total of 175 (53.2%) patients had only typical manifestations: 47 patients with Calabar swellings, 77 with eyeworm, and 51 patients with both Calabar swellings and eyeworm. In comparison, 154 (46.8%) had at least 1 atypical manifestation, of which 29 patients had manifestations spanning 2 different body systems (Figure 2). No patients presented with atypical manifestations spanning 3 or more organ systems. (In the following, citations preceded by S refer to Supplementary References in the Supplementary Data, “Literature Review.”)

Eight patients (5.2%) had heart failure signs [S11, S97, S155, S184, S208, S217, S221], of which 4 were caused by endomyocardial fibrosis [S97, S155, S184, S221]. Ten patients (6.5%) had respiratory system manifestations. Six were caused by pleural effusion [S39, S53, S74, S80, S228, S229]; 5 contained mf in the pleural fluid [S39, S53, S74, S228, S229], and 1 demonstrated mf on broncho-alveolar lavage (BAL) [S80]. Five of 6 patients had resolution of the pleural effusion after receiving antifilarial treatment [S39, S74, S228, S229]. Three patients had signs caused by pulmonary fibrosis [S72, S137, S186], with 1 patient demonstrating mf on BAL [S137]. One patient suffered from acute respiratory distress syndrome, although it was unclear if loiasis was responsible [S44].

Ten patients (6.5%) had gastrointestinal system manifestations [S6, S35, S49, S51, S70, S74, S100, S124]. Two patients had ascites [S74, S79]; 1 contained mf in the ascitic fluid, which resolved with antifilarial treatment [S79]. Microfilariae were incidentally discovered in 2 patients during a gastric [S6] and peritoneal lavage [S51]. Three patients had hypo-echogenic splenic lesions [S35, S100], of which 2 were associated with splenomegaly [S35]. One subsequent splenectomy demonstrated mf on spleen histology. One patient had bowel obstruction caused by constricting, thickened, and fibrotic lesion of the descending colon, containing mf [S124]. One patient had polyps containing mf upon resection [S70]. Finally, 1 patient suffered from acute hepatitis that improved with antifilarial therapy [S79].

Fifteen patients (9.7%) had renal system manifestations [S12, S62, S77, S89, S116, S149, S169, S192, S201, S224, S227, S234, S256, S258, S268], ranging in severity from asymptomatic proteinuria to severe nephrotic syndrome and end-stage renal failure. In the 8 cases where a renal biopsy was performed for the investigation of nephrotic syndrome [S77, S89, S116, S149, S224, S227, S234], membranous glomerulonephropathy was shown in all but 2 cases, in which focal interstitial inflammation [S227] and focal segmental glomerulosclerosis [S234] were shown. One patient suffered from renal failure secondary to interstitial fibrosis and sclerosis of the glomeruli [S89]. Four patients had microfilaruria (mf in urine [S12, S89, S256, S258]), and 3 had mf on histopathology analysis of their renal biopsy [S62, S224, S234].

Nine patients (5.8%) had reproductive system manifestations [S22, S38, S124, S131, S164, S220, S226]. Microfilariae were isolated from a sample of follicular fluid in 2 infertile women [S22, S226] and incidentally found in 3 women during a cervical smear [S38, S164, S220]. Three men were found to have an adult worm in their testicles (tunica vaginalis or spermatid cord) after orchidectomy due to testicular swelling [S124]. Seven (4.5%) women had mammogram results that were highly suggestive of the presence of a *L. loa* worm, reporting linear, straight, or curved calcifications 0.3–2.5 cm in length [S92, S117, S140, S166, S210, S216]. The indication for mammogram varied, with 4 undergoing routine screening, 2 for a painful breast lump, and 1 for bilaterally painful breasts. Two women had excisions performed [S117, S166], and in 1 an adult *L. loa* was isolated [S117].

Nineteen patients (12.3%) had joint-related manifestations [S8, S34, S37, S43, S103, S107, S120, S128, S135, S148, S150, S151, S174, S183, S193, S205, S232]. Five patients were assessed for an acute septic joint [S8, S34, S107, S128, S151]. Of the 4 patients whose joints were aspirated for articular fluid, 2 demonstrated mf in the synovial fluid [S107, S128], and the other 2 other showed eosinophilia between 60% and 80% [S34, S151]. Five patients demonstrated dead calcified worms on x-ray in painful wrist and finger joints [S37, S103, S120, S150]. Four patients experienced severe arthralgic joint pains that resolved with antifilarial treatment [S35, S43, S193, S232]. Two patients had an acute thrombotic event in the ulnar vein [S148] and popliteal artery [S205].

There were 25 cases (16.2%) related to the nervous system [S11, S14, S28, S32, S55, S57, S66, S90, S100, S158, S159, S167, S223, S225, S257, S260, S261, S262, S266, S268, S272]. There were 9 cases presenting with spontaneous encephalitis (before any antifilarial treatment) [S158, S167, S223, S257, S260, S261, S268], of which 8 cases had mf in the cerebrospinal fluid (CSF) [S158, S167, S223, S257, S261, S268]. Two patients had severe headaches refractory to medical treatment that resolved with antifilarial therapy [S14, S90]. There were 4 cases of ulnar nerve palsies [S55, S57, S100, S159] and 1 median nerve palsy whose neurological deficit resolved after antifilarial treatment [S57]. One patient had neck pain caused by cervical nerve root irritation with associated worms erupting from the overlying skin [S32]. One patient was diagnosed with an acute change in personality and psychotic polydipsia; the altered behavior resolved with antifilarial treatment [S272]. Three cases presented with dizziness, a change in hearing, or a change in fine motor skills, but there was no clear unifying diagnosis [S11, S28, S272].

There were 32 cases (20.8%) of ophthalmic manifestations outside the classical adult worm migration under the conjunctiva [S26, S42, S73, S78, S93, S95, S108, S115, S123, S130, S145, S169, S182, S187, S190, S211, S236, S250, S259, S266]. Twenty-one cases reported reduced/partial loss of vision [S73, S93, S123, S145, S169, S187, S190, S236, S259, S266], and 5 reported total (unilateral/bilateral) loss of vision [S26, S42, S78, S182, S211]. In the cases for which follow-up information was available, 9 patients had improvement in the loss of vision after treatment and 7 did not. There were 5 cases of reduced/blurred/loss of vision in which an adult worm was identified in the anterior chamber of the eye [S42, S78, S93, S145, S190]; 2 of these demonstrated an inflammatory membrane adherent to the anterior iris. An adult worm was seen in the posterior chamber in 1 case with loss of vision [S169]. Microfilariae were found in the anterior and posterior segments of the eye in 5 cases [S169, S259]. In the remaining cases with loss of vision where the worm was not successfully visualized by the physician, a variety of pathologies were described. These were chorioretinitis, corneal edema, posterior chamber opacification, vitreous hemorrhage, hemorrhagic retinopathy, retinal detachment, neovascularization, retinal artery occlusion, and uveitis. From the 32 patients with atypical ocular symptoms, there was no report of co-infections with other agents causing ocular manifestations such as *Onchocerca volvulus* and/or *Chlamydia trachomatis*, nor were onchocerciasis or trachoma mentioned as possible causes of the manifestations observed (Supplementary Data, “Atypical Ocular Manifestations,” Supplementary Table 1).

There were 20 cases (13.0%) of dermatological signs, with 8 cases of *L. loa* worms perforating through the skin (without any preceding antifilarial treatment) [S26, S95, S96, S98, S141, S154, S156, S222]. Two patients experienced skin swellings, 1 nasal [S162] that was excised to identify a worm. The remaining cases were skin rashes of varying nature: transient urticarial rashes, widespread exanthema rashes, and multiple pruritic lesions. One patient suffered from a vasculitis-like syndrome in which a biopsy of the purpuric rash demonstrated infiltrative eosinophils [S189].

Of the 199 cases of eosinophilia, 182 (91.5%) did not have any other co-infecting helminth reported. Of the 16 cases with (helminth) co-infections (8.5%), 8 presented with *Mansonella perstans*[S192, S217, S218, S227, S254, S263, S273, S274], 1 with *O. volvulus*[S225], 6 with soil-transmitted helminths (roundworm, whipworm, hookworm and threadworm) [S19, S198, S229, S260, S262, S272], and 2 with schistosomiasis [S232, S233] (Supplementary Data, “Eosinophilia and Co-Infections”).

### Factors Associated With Atypical Loiasis Manifestations

**Figure 3.**
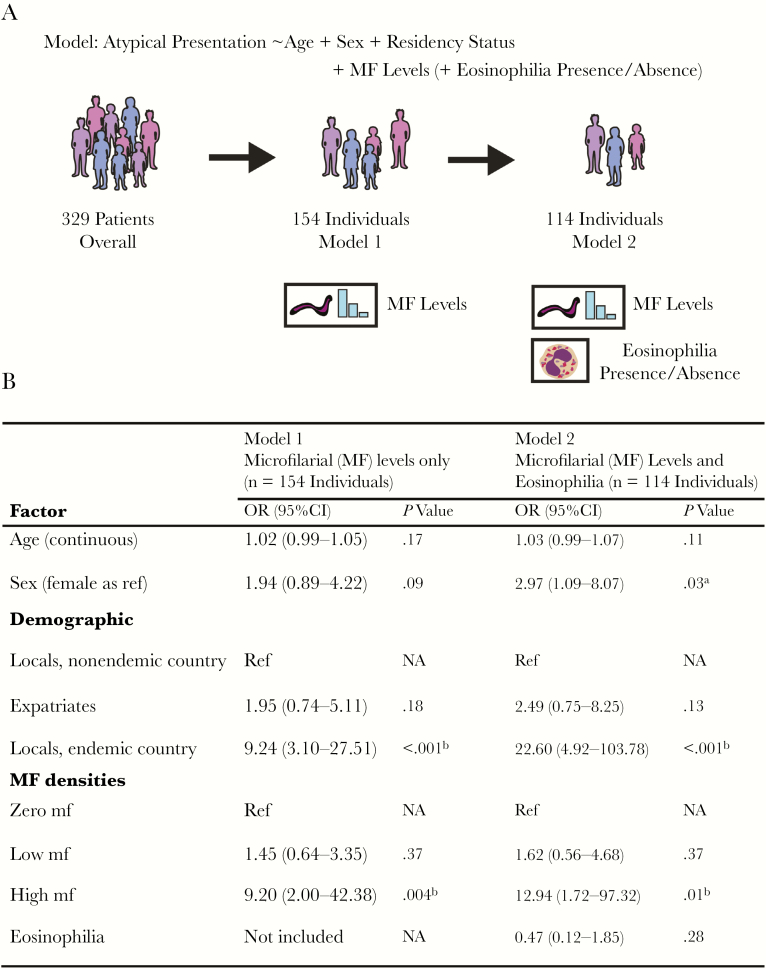
Association between individual-level factors and atypical loiasis presentation. Schematic illustration of the 2 multivariate logistic regression models used to quantify the influence of various factors on atypical loiasis presentation (A) and the output of these models (B). Microfilarial (MF) density levels were categorised as zero (0 mf/mL blood recorded), low (>0 but <8000 mf/mL blood), and high (≥8000 mf/mL blood). ^a^Indicates statistical significance or near significance (*P* ≤ .05). ^b^Indicates statistical significance (*P* ≤ .01). Abbreviations: CI, confidence interval; MF, microfilarial; mf, microfilariae; OR, odds ratio.

Information on microfilarial densities and typical/atypical manifestations was available for 172 patients (information on presence/absence of mf, but not on densities, was available for 276 patients). There was a statistically significant positive association between blood microfilarial density categories (amicrofilaremic, low and high) and atypical presentation (atypical or typical, *P* = .001, Pearson’s chi-square test) (Supplementary Data, “Statistical Analysis,” Supplementary Table 2). Based on this, 2 multivariate logistic regression models (Models 1 and 2, Figure 3A) were constructed to quantify the impact of microfilarial load on atypical presentation, controlling for demographic variables.

Model 1 had presentation status (typical/atypical) as the outcome variable and age, sex, residency status, and microfilarial density categories as covariates (n = 154 individuals). Model 2, a more restricted model, included individuals for whom the above information was available but who also had their eosinophilia status determined (n = 114 individuals). This second model was considered to control for the potential influence of eosinophilia status, suggesting, in light of recent findings, a role of eosinophils in pathologies arising from microfilarial infection in other filariae [20], as well as accounting for potential differences in eosinophil-associated processes between individuals indigenous to *L. loa–*endemic areas and those temporarily residing in such areas [21].

The results of both models demonstrated a positive association between blood microfilarial levels and atypical presentation (Figure 3B). Individuals with high microfilarial densities were more likely to present with atypical manifestations compared with amicrofilaremic individuals (Model 1: OR, 9.20; 95% CI, 2.00–42.38; *P* = .004; Model 2: OR, 12.94; 95% CI, 1.72–97.32; *P* = .01). Individuals with low microfilarial loads were not significantly more likely to present atypically compared with amicrofilaremic individuals (Model 1: *P* = .37; Model 2: *P* = .37). Analysis of Model 2, including eosinophilia status, indicated that eosinophilia was not significantly associated with the risk of presenting with atypical manifestations (OR, 0.47; 95% CI, 0.12–1.85; *P* = .28), although, interestingly, most individuals analyzed (86%) were eosinophilic (98/114).

**Figure 4.**
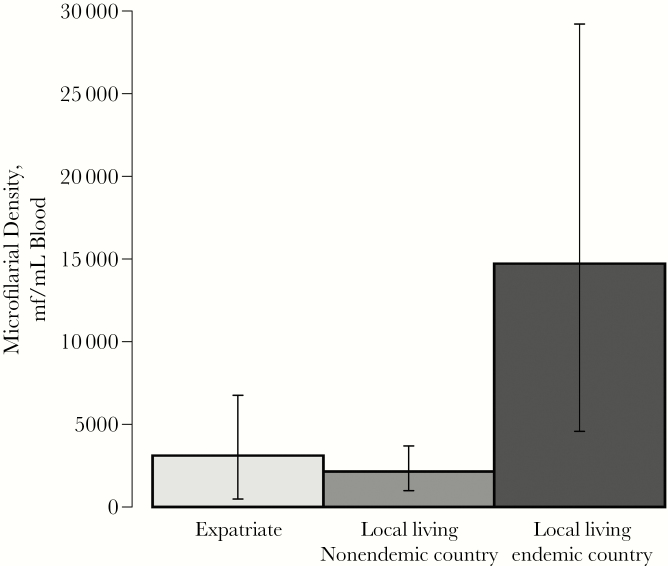
Average microfilarial densities for each of the residency categories examined in the analyses. The mean microfilarial densities for each residency group, locals residing in endemic countries (n = 40), locals residing in nonendemic countries (n = 44), and expatriates (n = 81) were calculated as the arithmetic means of the reported microfilarial densities, quantified by microscope blood smear. Error bars represent the 95% confidence intervals calculated using bootstrapping (see the Supplementary Data, “Confidence Interval Calculation: Non-Parametric Bootstrap”). Abbreviation: mf, microfilariae.

Both models revealed differences in the propensity for different residency status groups to present atypically. Locals residing in endemic countries were significantly more likely than locals residing in nonendemic countries and expatriates to have atypical manifestations (Figure 3B). This effect is in addition to and independent of microfilarial load, although it should be noted that locals residing in endemic countries also had the highest average microfilarial densities (Figure 4), further compounding their risk.

## DISCUSSION

Although our work corroborates findings from previous reviews regarding the variation in loiasis clinical profiles across different residency groups [14, 22], its size and focus on nontypical presentations provide novel insight into poorly resolved aspects of loiasis clinical epidemiology. Specifically, it highlights that the range of pathologies associated with *L. loa* infection is far broader than just Calabar swellings and eyeworm passage [23]. Although this may be due to a bias toward atypical manifestations in the reports analyzed here (well-known manifestations not meriting case reports) [24, 25], their breadth is striking, and our work provides an important summary of such presentations. The review identified numerous cases of vital organ involvement, such as heart, liver, respiratory, and renal systems associated with loiasis. In addition, 25/32 (78.1%) cases with atypical visual manifestations were associated with partial or total vision loss (without onchocerciasis and/or trachoma). Although we were unable to prove causality, the legitimacy of the association is supported by corroboration of mf presence in tissues other than blood, such as pleural and ascitic fluid, eye chambers, etc., and improvement or successful resolution of pathology upon antifilarial treatment (which would not have taken place were the mf found in these tissues simply the result of their migration to sites of abnormal anatomy). However, we acknowledge we do not, at present, fulfill all the criteria to support causality, particularly because of the scarcity of longitudinal studies [5]. The proportion of cases with dermatological presentations (13%) may seem somewhat small; however, as the review focused on signs rather than symptoms, it likely excluded cases of itching (not systematically recorded in the reports).

These atypical conditions are more common than previously perceived, with 47% of examined patients presenting with a manifestation(s) outside the classical Calabar swellings and eyeworm. Individuals residing in endemic countries were at the greatest risk of developing atypical manifestations. The results presented here likely represent an overestimation of the true prevalence of atypical manifestations due to (various sources of) bias (eg, locals familiar with classical signs may only consult if suffering from other symptoms, whereas expatriates/travelers may consult at the first sign of more typical manifestations). However, our aim was not to evaluate the true proportion of atypical manifestations of loiasis but to describe and summarize the breadth of these presentations and, importantly, to evaluate whether they were related to *L. loa* microfilarial density. Thus, although we acknowledge recruitment bias in the case reports, this does not impact the analyses performed, as patients did not know their microfilarial density at the time of consultation. We advocate that large-scale surveys (eg, [10]) be conducted to estimate rigorously the rates of atypical presentations in endemic areas. Notwithstanding these limitations, even a smaller percentage of individuals experiencing atypical manifestations of loiasis would represent a substantial number of people given that 14 million reside in areas of high loiasis prevalence and intensity in Central Africa [2].

The analyses presented here also highlight that individuals with high microfilarial densities are at the greatest risk of suffering atypical manifestations. Although an association between infection intensity and disease is typically assumed in parasitic infections, its demonstration is essential to link infection to morbidity and mortality. Further work (with larger sample sizes and greater statistical power) is needed to corroborate this association (and to identify any dose–response relationship with microfilarial density). Heavy infection intensity was identified as a crucial factor in a recent study examining the influence of *L. loa* microfilarial infection on increased risk of mortality [5]. Although the mechanism underpinning this increased risk remains unclear, possibilities include obstructive processes associated with parasite carriage [26] and/or more general inflammatory responses inducing pathogenic processes in various vital organs. León et al. [27] identified endomyocardial fibrosis in a patient from an endemic area who had *L. loa* microfilaremia and marked eosinophilia.

As high microfilarial densities also increase the probability of SAEs after ivermectin treatment [8], targeted treatment of individuals most at risk may require drugs that are not strongly and/or fast-acting microfilaricidal. Evidence supports the use of albendazole in instances where other anthelmintics would be unsafe [28, 29], although questions remain surrounding the efficacy and optimal dosing regimen [30].

For those with lower microfilarial densities, ivermectin is highly efficacious at clearing *L. loa* mf [31]. Recent studies in areas co-endemic for loiasis and onchocerciasis evaluating the impact of mass ivermectin treatment on microfilarial prevalence and intensity of loiasis have yielded promising results in terms of reductions in the prevalence of heavy microfilaremia [32] (although others have reported less clear-cut results on entomological indicators of transmission [33]). However, not all areas endemic for loiasis are also co-endemic with onchocerciasis and/or lymphatic filariasis [34, 35], and thus would not receive ivermectin or ivermectin plus albendazole treatment for these filariases. Hence, perhaps with the exception of those loiasis-only areas in which control programs for soil-transmitted helminthiases are being implemented using benzimidazole anthelmintics (to which albendazole belongs), our results suggest that substantial reductions in the burden of loiasis in these communities could be achieved through campaigns targeting loiasis directly (through chemotherapeutic and/or tabanid control interventions) [1, 28–32, 36].

## CONCLUSIONS

The work presented here suggests that conceptions of loiasis as a relatively benign infection are likely misplaced and emphasizes the public health importance of loiasis. Our results highlight the wide spectrum of clinical conditions associated with *L. loa* carriage, including visual impairment and blindness (typically disregarded as sequelae of loiasis). Although estimates of the burden of disease attributable to loiasis remain outstanding, these will likely be significantly higher than anticipated. This will be especially true in communities bearing high microfilarial burdens, as severe manifestations of loiasis occur at higher frequencies in heavily infected individuals. We advocate that large-scale, community-wide (and ideally longitudinal) studies be conducted in endemic regions to ascertain the true prevalence of atypical loiasis manifestations and the association between infection intensity and morbimortality to better understand the clinical and public health burden of loiasis in its own right and not merely as an impediment to the control of other filarial diseases [37].

## Supplementary Data

Supplementary materials are available at *Open Forum Infectious Diseases* online (*https://academic.oup.com/ofid*). Consisting of data provided by the authors to benefit the reader, the posted materials are not copyedited and are the sole responsibility of the authors, so questions or comments should be addressed to the corresponding author.

ofz417_suppl_Supplementary-File

## References

[1] Whittaker C, Walker M, Pion SDS, et al. The population biology and transmission dynamics of *Loa loa*. Trends Parasitol 2018; 34:335–50. 29331268 10.1016/j.pt.2017.12.003

[2] Zouré HG, Wanji S, Noma M, et al. The geographic distribution of *Loa loa* in Africa: results of large-scale implementation of the Rapid Assessment Procedure for Loiasis (RAPLOA). PLoS Negl Trop Dis 2011; 5:e1210. 21738809 10.1371/journal.pntd.0001210 PMC3125145

[3] Pinder M *Loa loa* - a neglected filaria. Parasitol Today 1988; 4:279–84. 15463001 10.1016/0169-4758(88)90019-1

[4] World Health Organization. Neglected tropical diseases 2018 Available at: http://www.who.int/neglected_diseases/diseases/en/. Accessed 30 November 2018.

[5] Chesnais CB, Takougang I, Paguélé M, et al. Excess mortality associated with loiasis: a retrospective population-based cohort study. Lancet Infect Dis 2017; 17:108–16. 27777031 10.1016/S1473-3099(16)30405-4

[6] Fischer PU Filarial infection deserves attention as neglected tropical disease. Lancet Infect Dis 2017; 17:12–3. 27777032 10.1016/S1473-3099(16)30382-6

[7] Hoerauf A, Pfarr K, Mand S, et al. Filariasis in Africa—treatment challenges and prospects. Clin Microbiol Infect 2011; 17:977–85. 21722251 10.1111/j.1469-0691.2011.03586.x

[8] Gardon J, Gardon-Wendel N, Demanga-Ngangue, et al. Serious reactions after mass treatment of onchocerciasis with ivermectin in an area endemic for *Loa loa* infection. Lancet 1997; 350:18–22. 9217715 10.1016/S0140-6736(96)11094-1

[9] Boussinesq M, Gardon J, Gardon-Wendel N, et al. Three probable cases of *Loa loa* encephalopathy following ivermectin treatment for onchocerciasis. Am J Trop Med Hyg 1998; 58:461–9. 9574793 10.4269/ajtmh.1998.58.461

[10] Noireau F, Apembet JD, Nzoulani A, Carme B Clinical manifestations of loiasis in an endemic area in the Congo. Trop Med Parasitol 1990; 41:37–9. 2339244

[11] Akue JP, Nkoghe D, Padilla C, et al. Epidemiology of concomitant infection due to *Loa loa* and *Mansonella perstans* in Gabon. PLoS Negl Trop Dis 2011; 5:e1329. 22022623 10.1371/journal.pntd.0001329 PMC3191124

[12] Gobbi F, Postiglione C, Angheben A, et al. Imported loiasis in Italy: an analysis of 100 cases. Travel Med Infect Dis 2014; 12:713–7. 25131142 10.1016/j.tmaid.2014.07.004

[13] Gantois N, Rapp C, Gautret P, et al. Imported loiasis in France: a retrospective analysis of 47 cases. Travel Med Infect Dis 2013; 11:366–73. 24035648 10.1016/j.tmaid.2013.08.005

[14] Churchill DR, Morris C, Fakoya A, et al. Clinical and laboratory features of patients with loiasis (*Loa loa* filariasis) in the U.K. J Infect 1996; 33:103–9. 8889997 10.1016/s0163-4453(96)93005-4

[15] Saito M, Armstrong M, Boadi S, et al. Clinical features of imported loiasis: a case series from the hospital for tropical diseases, London. Am J Trop Med Hyg 2015; 93:607–11. 26101271 10.4269/ajtmh.15-0214 PMC4559705

[16] Delabre S, Parola P, Thiberville D, et al. Non-ophthalmological presentation of imported loiasis. Travel Med Infect Dis 2014; 12:406–9. 24857349 10.1016/j.tmaid.2014.04.012

[17] Johann C, Eric C, Sophie M, et al. Imported loiasis in France: a 10 years retrospective review with comparison between African migrants and non-African travelers. Trop Med Int Health 2013; 18(Suppl 1):43.

[18] Develoux M, Hennequin C, Le Loup G, et al. Imported filariasis in Europe: a series of 31 cases from Metropolitan France. Eur J Intern Med 2017; 37:e37–9. 27733303 10.1016/j.ejim.2016.09.021

[19] Valent P, Klion AD, Horny HP, et al. Contemporary consensus proposal on criteria and classification of eosinophilic disorders and related syndromes. J Allergy Clin Immunol 2012; 130:607–612.e9. 22460074 10.1016/j.jaci.2012.02.019 PMC4091810

[20] Cadman ET, Thysse KA, Bearder S, et al. Eosinophils are important for protection, immunoregulation and pathology during infection with nematode microfilariae. PLoS Pathog 2014; 10:e1003988. 24626328 10.1371/journal.ppat.1003988 PMC3953434

[21] Herrick JA, Metenou S, Makiya MA, et al. Eosinophil-associated processes underlie differences in clinical presentation of loiasis between temporary residents and those indigenous to *Loa*-endemic areas. Clin Infect Dis 2015; 60:55–63. 25234520 10.1093/cid/ciu723 PMC4296126

[22] Antinori S, Schifanella L, Million M, et al. Imported *Loa loa* filariasis: three cases and a review of cases reported in non-endemic countries in the past 25 years. Int J Infect Dis 2012; 16:e649–62. 22784545 10.1016/j.ijid.2012.05.1023

[23] Agbolade OM, Akinboye DO, Ogunkolo OF *Loa loa* and *Mansonella perstans*: neglected human infections that need control in Nigeria. Afr J Biotechnol 2005; 4:1554–8.

[24] Carme B, Mamboueni JP, Copin N, Noireau F Clinical and biological study of *Loa loa* filariasis in Congolese. Am J Trop Med Hyg 1989; 41:331–7. 2679158

[25] Nutman TB, Miller KD, Mulligan M, Ottesen EA *Loa loa* infection in temporary residents of endemic regions: recognition of a hyperresponsive syndrome with characteristic clinical manifestations. J Infect Dis 1986; 154:10–8. 3458832 10.1093/infdis/154.1.10

[26] Corrigan MJ, Hill DW Retinal artery occlusion in loiasis. Br J Ophthalmol 1968; 52:477–80. 5665947 10.1136/bjo.52.6.477 PMC506625

[27] León D, Martín M, Corros C, et al. Usefulness of cardiac MRI in the early diagnosis of endomyocardial fibrosis. Rev Port Cardiol 2012; 31:401–2. 22503434 10.1016/j.repc.2011.12.016

[28] Klion AD, Massougbodji A, Horton J, et al. Albendazole in human loiasis: results of a double-blind, placebo-controlled trial. J Infect Dis 1993; 168:202–6. 8515109 10.1093/infdis/168.1.202

[29] Kamgno J, Boussinesq M Effect of a single dose (600 mg) of albendazole on *Loa loa* microfilaraemia. Parasite 2002; 9:59–63. 11938697 10.1051/parasite/200209159

[30] Kamgno J, Nguipdop-Djomo P, Gounoue R, et al. Effect of two or six doses 800 mg of albendazole every two months on *Loa loa* microfilaraemia: a double blind, randomized, placebo-controlled Trial. PLoS Negl Trop Dis 2016; 10:e0004492. 26967331 10.1371/journal.pntd.0004492 PMC4788450

[31] Pion SDS, Tchatchueng-Mbougua JB, Chesnais CB, et al. Effect of a single dose of ivermectin on *Loa loa* microfilaremia: systematic review and meta-analysis. Open Forum Infect Dis 2019; 6(X):XXX–XX. 10.1093/ofid/ofz019 PMC6449757 30968052

[32] Wanji S, Chounna Ndongmo WP, Fombad FF, et al. Impact of repeated annual community directed treatment with ivermectin on loiasis parasitological indicators in Cameroon: implications for onchocerciasis and lymphatic filariasis elimination in areas co-endemic with *Loa loa* in Africa. PLoS Negl Trop Dis 2018; 12:e0006750. 30226900 10.1371/journal.pntd.0006750 PMC6161907

[33] Kouam MK, Tchatchueng-Mbougua JB, Demanou M, et al. Impact of repeated ivermectin treatments against onchocerciasis on the transmission of loiasis: an entomologic evaluation in central Cameroon. Parasit Vectors 2013; 6:283. 24289520 10.1186/1756-3305-6-283 PMC3849770

[34] Cano J, Basáñez MG, O’Hanlon SJ, et al. Identifying co-endemic areas for major filarial infections in sub-Saharan Africa: seeking synergies and preventing severe adverse events during mass drug administration campaigns. Parasit Vectors 2018; 11:70. 29382363 10.1186/s13071-018-2655-5 PMC5791223

[35] Vinkeles Melchers NVS, Coffeng LE, Boussinesq M, et al Projected number of people with onchocerciasis-loiasis co-infection in Africa, 1995 to 2025. Clin Infect Dis. In press. 10.1093/cid/ciz647 PMC7245158 31304961

[36] Kelly-Hope L, Paulo R, Thomas B, et al. *Loa loa* vectors *Chrysops* spp.: perspectives on research, distribution, bionomics, and implications for elimination of lymphatic filariasis and onchocerciasis. Parasit Vectors 2017; 10:172. 28381279 10.1186/s13071-017-2103-y PMC5382514

[37] Metzger WG, Mordmüller B *Loa loa*—does it deserve to be neglected? Lancet Infect Dis 2014; 14:353–7. 24332895 10.1016/S1473-3099(13)70263-9

