# Supplementary material for: Atypical Clinical Manifestations of Loiasis and Their Relevance for Endemic Populations
Source: Open Forum Infect Dis. 2019 Nov 1;6(11):ofz417. doi: 10.1093/ofid/ofz417 (PMC6824532; doi:10.1093/ofid/ofz417)
Supplement: ofz417_suppl_Supplementary-File [file ofz417_suppl_supplementary-file.pdf]

Supplementary File

Atypical Clinical Manifestations of Loiasis and their Relevance for Endemic Populations.

Kevin G. Buell[1*§], Charles Whittaker[1*], Cédric B. Chesnais[2], Paul D. Jewell[1], Sébastien D.S. Pion[2], Martin Walker[3], Maria-Gloria Basáñez[1¶,] Michel Boussinesq[2¶]

[1] Department of Infectious Disease Epidemiology, London Centre for Neglected Tropical Disease Research and MRC Centre for Global Infectious Disease Analysis, Faculty of Medicine (St Mary's Campus), Imperial College London, London W2 1PG, UK.

[2] Institut de Recherche pour le Développement (IRD), UMI 233-INSERM U1175-Montpellier University, Montpellier, France.

[3] Department of Pathobiology and Population Sciences and London Centre for Neglected Tropical Disease Research, Royal Veterinary College, Hatfield, UK.

[*] Contributed equally to this work. [§] Present address: Department of Medicine, Vanderbilt University Medical Center, 1211 Medical Center Drive, Nashville, TN 37232, USA. [¶] Equal senior authors.

Correspondence to:
Prof María-Gloria Basáñez
Department of Infectious Disease Epidemiology
London Centre for NTD Research
Imperial College London
St Mary's campus
Norfolk Place
London, W2 1PG, UK
Office: +44 20 7594 3295
m.basanez@imperial.ac.uk

Alternative contact:
Dr Michel Boussinesq
Institut de Recherche pour le Développement
Unité Mixte Internationale (UMI) 233
Institut National de la Santé et de la Recherche Médicale (INSERM) U1175
Université de Montpellier
911 Avenue Agropolis
34394 Montpellier Cedex 5, France
Office: +33 4 67 41 64 41
michel.boussinesq@ird.fr

Supplementary Methods

1. Literature Review (Supplementary References)

Below is the full list of papers included in the study following the systematic literature review.

S1. Aiello F, Palma S, Varesi C, Cerulli A, Valente R, Aiello L. A rare case report of *Loa loa* ocular filariasis. Eur J Ophthalmol. **2010;** 20(1): 237-239.

S2. Ali S, Fisher M, Juckett G. The African eye worm: a case report and review. J Travel Med. **2008;**15(1): 50-52.

S3. Baartman BJ, Nguyen L, Wiest P, Steinemann TL. Ocular loiasis in Ohio: a case report. Int Ophthalmol. **2018;** 38(5): 2167-2170.

S4. Berger SA, Sigman-Igra Y, Geyer O, Michaeli D, Lengy J. Filariasis in Israel. Isr J Med Sci. **1988;** 24(11): 690-691.

S5. Abdou AMS, Abro AH, Younas NJ, Ustadi AM, Sultan DM. *Loa loa* infection in non-endemic area: a case study and disease review. Emirates Med J. **2008;** 26(3): 159-163.

S6. Whitaker D, Reed WD, Shilkin KB. A case of filariasis diagnosed on gastric cytology. Pathology. **1980;** 13(3): 483-486.

S7. Felts WR, Talbott EJ. A study of host-parasite relationship in *Loa loa*; a case report. Am J Med. **1957;** 22(6): 995-1003.

S8. Low K, Barker AJ, Vincent JM. A thirteen-year-old with a painful, swollen foot and eosinophilia. Pediatr Infect Dis J. **1995;** 14(9): 820, 825-826.

S9. Stretch R. A treatment for loiasis. Nurs Times. **1999;** 95(33): 50-51.

S10. Rivière E, Kerautret J, Combillet F, Malvy D. African eye worm. J Glob Infect Dis. **2012;** 4(2): 135-136.

S11. Alajouanine TH, Castaigne P, Lhermitte F, Gambier J. [Encephalitis followed by fibroblastic endocarditis of filarian origin]. Rev Neurol (Paris). **1959;** 101(5): 656-660 (in French).

S12. Bariéty J, Barbier M, Laigre MC, Tchernia G, Lagrue G, Samarcq P, *et al.* [Proteinuria and loaiasis. Histologic, optic and electronic study of a case]. Bull Mem Soc Med Hop Paris. **1967;** 118(11): 1015-1025 (in French).

S13. Barnett JM, Wolter JR. *Loa loa*: the African eye worm observed in Michigan. J Pediatr Ophthalmol. **1971;** 8(1): 23-25.

S14. Bickerstaff ER. Allergic basis for migraine: a lesson from *Loa loa*. Br Med J. **1957;** 1(5014): 327.

S15. Bowler GS, Shah AN, Bye LA, Saldana M. Ocular loiasis in London 2008-2009: a case series. Eye (Lond). **2011;** 25(3): 389-391.

S16. Brain RT. Loa-Loa. Proc R Soc Med. **1931;** 24(5): 515-516.

S17. Brice P, Etienne SD, Le Thi Huong Du, Wechsler B, Chandenier J, Piette JC, *et al.* [*Loa loa* filariasis, encephalitis and treatment with notézine: an underestimated complication?]. Ann Med Interne (Paris). **1989;** 140(4): 319-320 (in French).

S18. Brown IA. *Loa loa* infestation in an immigrant to Britain. Proc R Soc Med. **1968;** 61(6): 551.

S19. Ten Berg JA. [Hetrazan therapy of *Loa* infection]. Ned Tijdschr Geneeskd. **1952;** 96(39): 2411-2417 (in Dutch).

S20. Yoshikawa M, Ouji Y, Hayashi N, Moriya K, Nishiofuku M, Ishizaka S, *et al.* Diagnostic problems in a patient with amicrofilaremic *Loa loa*. J Travel Med. **2008;** 15(1): 53-57.

S21. Workman DMS, McNab AA. *Loa loa* disease. Aust N Z J Ophthalmol. **1990;** 18(3): 357-358.

S22. Wisanto A, Laureys M, Camus M, Devroey P, Verheyen G, Van Steirteghem AC. Case report: *Loa loa* microfilariae aspirated during oocyte retrieval. Hum Reprod. **1993;** 8(12): 2096-2097.

S23. Wiesinger EC, Winkler S, Egger S, Burgmann H, Graninger W. [Worm in the eye as first manifestation of an infection with *Loa loa*]. Dtsch Med Wochenschr. **1995;** 120 (34-35): 1156-1160 (in German).

S24. Weitzel T, Jelinek T. Images in clinical medicine. Loiasis. N Engl J Med. **2006;** 355(7): e6.

S25. Wang X, Zhang X, Zong Z. A case of loiasis in a patient returning to China diagnosed by nested PCR using DNA extracted from tissue. J Travel Med. **2012;** 19(5): 314-316.

S26. de Viragh PA, Guggisberg D, Derighetti M, van Saanen M, Panizzon RG. Monosymptomatic *Loa loa* infection. Dermatology. **1998;** 197(3): 303-305.

S27. Reisman J, Krolman GM, Hogg GR. Conjunctival *Loa loa*. Can J Ophthalmol. **1974;** 9(3): 379-381.

S28. Veit O, Beck B, Steuerwald M, Hatz C. First case of ivermectin-induced severe hepatitis. Trans R Soc Trop Med Hyg. **2006;** 100(8): 795-797.

S29. Varhaug P. Subconjunctival *Loa loa*: first case report from Norway. Acta Ophthalmol (Oxf). **2009;** 87(8): 929-930.

S30. Saeed AA, Green PJ, Naoroz M, Lee HA, Raman GV. *Loa loa* - the use of a blood-cell separator to reduce microfilaremia before specific chemotherapy. J Infect. **1984;** 9(2): 161-166.

S31. Saeed AA, Lee H. Usefulness of apheresis to extract microfilarias in management of loiasis. Br Med J (Clin Res Ed). **1983;** 287(6400): 1223.

S32. Browne SG. [Bilateral herpes zoster due to *Loa loa*]. Ann Soc Belg Med Trop. **1954;** 34(1): 5-8 (in French).

S33. Brumpt LC, Cornu P, Jaeger G, Neveu JY, Parc R. [Loaiasis with high microfilaremia. Attempted extracorporal purification after exsanguination-transfusion]. Bull Soc Pathol Exot Filiales. **1969;** 62(5): 900-907 (in French).

S34. Buchard PA, Gerster JC. [What is your diagnosis? Reactive arthritis due to loa filaria (*Loa loa*)]. Schweiz Rundsch Med Prax. **1990;** 79(16): 479-480 (in French).

S35. Burchard GD, Reimold-Jehle U, Burkle V, Kretschmer H, Vierbuchen M, Racz P, *et al.* Splenectomy for suspected malignant lymphoma in two patients with loiasis. Clin Infect Dis. **1996;** 23(5): 979-982.

S36. Charters AD, Welborn TA, Miller P. Calabar swellings in immigrants in Western Australia. Med J Aust. **1972;** 1(6): 268-271.

S37. Williams I. Calcification in loiasis. J Fac Radiol. **1954;** 6(2): 142-144.

S38. Callihan TR, Oertel YC, Mendoza M. *Loa loa* in a gynecologic smear. Am J Trop Med Hyg. **1977;** 26(3): 572-573.

S39. Cambanis A. Pulmonary loiasis and HIV coinfection in rural Cameroon. PLoS Negl Trop Dis. **2010;** 4(3): e572.

S40. Carbonez G, Van De Sompel W, Zeyen T. Subconjunctival *Loa loa* worm: case report. Bull Soc Belge Ophtalmol. **2002;** 283: 45-48.

S41. Johnson GJ, Axsmith K, Desser SS. The elusive *Loa loa*. A case report of ocular filariasis in Canada. Can J Ophthalmol. **1973;** 8(3): 492-496.

S42. Carme B, Kaya-Gandziami G, Pintart D. [Localization of the filaria *Loa loa* in the anterior chamber of the eye. Apropos of a case]. Acta Trop. **1984;** 41(3): 265-269 (in French).

S43. Carme B, Nkoua JL. [*Loa loa* filariasis: a cause of severe hypereosinophilia]. Bull Soc Pathol Exot Filiales. **1989;** 82(4): 581-583 (in French).

S44. Chani M, Iken M, Eljahiri Y, Nzenze JR, Mion G. [Acute respiratory distress syndrome caused by tropical eosinophilic lung disease: a case in Gabon]. Med Trop (Mars). **2011;** 71(2): 181-182 (in French).

S45. Cho HY, Lee YJ, Shin SY, Song HO, Ahn MH, Ryu JS. Subconjuctival *Loa loa* with Calabar swelling. J Korean Med Sci. **2008;** 23(4): 731-733.

S46. Ciobotaru M, Ephros M, Bitterman H. Filariasis caused by *Loa loa* in an Israeli temporary resident of West Africa. Isr J Med Sci. **1993;** 29(4): 219-221.

S47. Kern P, Wind P, Dietrich M. Continuous-flow apheresis of microfilariae in *Loa loa* infestations. Transfus Apher Sci. **1988;** 9(1): 95-98.

S48. Corrigan MJ, Hill DW. Retinal artery occlusion in loiasis. Br J Ophthalmol. **1968;** 52(6): 477-480.

S49. Cruel T, Arborio M, Schill H, Neveux Y, Nedelec G, Chevalier B, *et al.* [Nephropathy and filariasis from *Loa loa*. Apropos of 1 case of adverse reaction to a dose of ivermectin]. Bull Soc Pathol Exot. **1997;** 90(3): 179-181 (in French).

S50. De Silva DJ, Strouthidis NG, Tariq S, Davies N. An unusual cause of acute lid swelling. Eye (Lond). **2006;** 20(2): 271-272.

S51. Bernal-Green LM. Detection of *Loa loa* microfilariae in peritoneal washings. Clin Microbiol Newsl. **1989;** 11(24): 190-191.

S52. Echeverría Irigoyen MJ, Cosme Jiménez A, García-Arenzana JM, González García A. Diagnostic utility of phase-contrast microscopy for filariasis: a case of loaiasis. Clin Microbiol Newsl. **2005;** 27(21): 169-170.

S53. Madell SH, Spingarn GL. Unusual thoracic manifestations in filariasis due to *Loa loa*; results of treatment with hetrazan and naphuride sodium. Am J Med. **1953;** 15(2): 272-280.

S54. Tyrrell E. A case of filaria loa. Lancet. **1919;** 194(5021): 946.

S55. Gobbi F, Angheben A, Mascarello M, Gobbo M, Rossanese A, Anselmi M, *et al.* Unusual clinical presentation of loiasis: A case report. Trop Med Int Health. **2009;** 14(Suppl

2): 171 (Abstract, 6th European Congress on Tropical Medicine and International Health and 1st Mediterranean Conference on Migration and Travel Health. Verona, Italy. September 6-10, 2009).

S56. Tyagi P, Asensio M, Bekir OA, Jabir M. Subconjunctival *Loa loa*. BMJ Case Rep. **2011;** pii: bcr0620103075.

S57. Schofield FD. Two cases of loiasis with peripheral nerve involvement. Trans R Soc Trop Med Hyg. **1955;** 49(6): 588-589.

S58. Bordon LM, Maurice M. Traveller's loiasis in Zimbabwe: a case report. Cent Afr J Med. **1994;** 40(11): 323-327.

S59. Thompson C, Cy A, Boggild AK. Chronic symptomatic and microfilaremic loiasis in a returned traveller. Can Med Assoc J. **2015;** 187(6): 437.

S60. Thompson JH. ACTH as an adjunct to the treatment of loaiasis. Am J Trop Med Hyg. **1956;** 5(6): 1103-1105.

S61. O'Donnell D, O'Connor L, Atherton P. There's a worm in my eye. Med J Aust. **1992;** 157(11-12): 833-834.

S62. Hocqueloux L, Kerdraon R, Niang M, Grézard O. The case | A Senegalese man with gross hematuria. Kidney Int. **2009;** 75(1): 125-126.

S63. Doan NM, Keiser PB, Bates RA, Fedorko DP, Weina PJ, Lucey DR. A 33-year-old woman from Nigeria with eosinophilia - Loiasis. Clin Infect Dis. **2002;** 35(10): 1204, 1263-1264.

S64. Eballe AO, Epée E, Koki G, Owono D, Mvogo CE, Bella AL. Intraocular live male filarial *Loa loa* worm. Clin Ophthalmol. **2008;** 2(4): 965-967.

S65. El Mellaoui M, El Ouafi A, Khalloufi A, Iferkhas S, Laktaoui A. [Subconjunctival loiasis: a case report]. Presse Med. **2015;** 44(7-8): 867-868 (in French).

S66. Elliot RH. Removal of worm (Filaria Loa) from the eye. Br Med J. **1918;** 1(2992): 502-504.

S67. Eveland LK, Yermakov V, Kenny M. *Loa loa* infection without microfilaraemia. Trans R Soc Trop Med Hyg. **1975;** 69(3): 354-355.

S68. Culpan V. Filariasis in an immigrant worker. Occup Health (Lond). **1973;** 25(1): 31.

S69. Goldsmid JM, Nightingale R, Clark D. Imported filarial infections in Tasmania. Med J Aust. **1980;** 1(13):667.

S70. Mougeot G, Marteau M, Riou C. [Discovery of many microfilariae *Loa loa* in sigmoid biopsy]. Bull Soc Franc Parasitol. **1985;** 2: 81 (in French).

S71. Ameur A, El Haouri M, Touiti D, Beddouch A, Oukheira H. [Hematuria and filariasis: pathogenic aspects: a case report]. Ann Urol (Paris). **2000;** 34(2): 110-111 (in French).

S72. Dickson RC. Loaiasis. Proc Mine Med Off Assoc. **1971;** 51(410): 136-139.

S73. Garin JP, Rougier J, Mojon M. [Loaiasis and posterior uveitis. Apropos of a case]. Acta Trop. **1975;** 32(4): 384-388 (in French).

S74. Ghys C, Morissens M, Rozen L, Karmali R, Theunissen C. Loiasis with pleural and peritoneal involvement. J Travel Med. **2012;** 19(3): 186-188.

S75. Giardulli A, Paulo Filho GD, Colombini GN, Eyer-Silva W de A, Basilio-de-Oliveira CA. A historical note on an imported case of loiasis in Rio de Janeiro, Brazil, 1964. Rev Inst Med Trop Sao Paulo. **2011;** 53(5): 295-297.

S76. Gupta A, Kedhar S. Images in clinical medicine. Eye worm. N Engl J Med. **2005;** 353(25): e22.

S77. Hall CL, Stephens L, Peat D, Chiodini PL. Nephrotic syndrome due to loiasis following a tropical adventure holiday: a case report and review of the literature. Clin Nephrol. **2001;** 56(3): 247-250.

S78. Hassan S, Isyaku M, Yayo A, Fada FS, Ihesiulor GU, Iliyasu G. Adult *Loa loa* filarial worm in the anterior chamber of the eye: a first report from savanna belt of northern Nigeria. PLoS Negl Trop Dis. **2016;** 10(4): e0004436.

S79. Hautekeete ML, Pialoux G, Marcellin P, Girard PM, Degott C, Benhamou JP. Presence of *Loa loa* microfilariae in ascitic fluid. J Infect Dis. **1989;** 160(3): 559-560.

S80. Hulin C, Rabaud C, May T, Neimann L, Kures L, Canton P. [Pulmonary involvement with a favorable course during *Loa loa* filariasis]. Bull Soc Pathol Exot. **1994;** 87(4): 248-250 (in French).

S81. Moffett S, Wills CP. Images in emergency medicine. Young man with foreign-body sensation in the right eye. Loaiasis (African eye worm). Ann Emerg Med. **2010;** 55(6): 578, 583.

S82. Antinori S, Schifanella L, Million M, Galimberti L, Ferraris L, Mandia L, *et al.* Imported *Loa loa* filariasis: three cases and a review of cases reported in non-endemic countries in the past 25 years. Int J Infect Dis. **2012;** 16(9): e649-e662.

S83. Recio R, Herrero-Martínez JM, Lizasoain M, Oliveira E, Pérez-Ayala A. Incidental finding observed on a blood smear. Clin Microbiol Infect. **2018;** 24(2): 145.

S84. Jain R, Chen JY, Butcher AR, Casson R, Selva D. Subconjunctival *Loa loa* worm. Int J Infect Dis. **2008;** 12(6): e133-e135.

S85. James RR, Hunt EL. Notes on a case of filaria loa (subconjunctival). Lancet. **1919;** 194(5020): 874.

S86. Jazuli F, Kelton TJ, Keystone JS. A diagnostic challenge: eosinophilia of unknown etiology. Travel Med Infect Dis. **2016;** 14(5): 537-538.

S87. Jolly BT, Foley KA. Loiasis: a case of an unusual ocular foreign body. Ann Emerg Med. **1992;** 21(9): 1153-1156.

S88. Kagmeni G, Cheuteu R, Bilong Y, Wiedemann P. Anterior chamber live *Loa loa*: case report. Clin Med Insights Case Rep. **2016;** 9: 55-56.

S89. Katner H, Beyt BE Jr, Krotoski WA. Loiasis and renal failure. South Med J. **1984;** 77(7): 907-908.

S90. Kazacos KR, Smith LE Jr. Loaiasis (*Loa loa*) in an African student in Indiana. Am J Trop Med Hyg. **1979;** 28(2): 213-215.

S91. Klion AD, Horton J, Nutman TB. Albendazole therapy for loiasis refractory to diethylcarbamazine treatment. Clin Infect Dis. **1999;** 29(3): 680-682.

S92. Lemmenmeier E, Keller N, Chuck N. Calcification of the breasts due to loiasis. IDCases. **2016;** 4: 8-9.

S93. Barua P, Barua N, Hazarika NK, Das S. *Loa loa* in the anterior chamber of the eye: a case report. Indian J Med Microbiol. **2005;** 23(1): 59-60.

S94. Grigsby ME, Keller DH. *Loa loa* in the District of Columbia. A case report. J Natl Med Assoc. **1971;** 63(3): 198-201.

S95. Sayali B, Baile RB, Snehal N, Gayatri J, Pratik G. *Loa loa* macrofilariasis in the eyelid: case report of the first periocular subcutaneous manifestation in India. J Parasit Dis. **2011;** 35(2): 230-231.

S96. Sbeity ZH, Jaksche A, Martin S, Loeffler KU. *Loa loa* macrofilariasis in the eyelid: case report of the first periocular subcutaneous manifestation in Germany. Graefes Arch Clin Exp Ophthalmol. **2006;** 244(7): 883-884.

S97. Andy JJ, Bishara FF, Soyinka OO, Odesanmi WO. Loasis as a possible trigger of African endomyocardial fibrosis: a case report from Nigeria. Acta Trop. **1981;** 38(2): 179-186.

S98. Lakshmi N, Gururaj KA. Loiasis (a case report). Indian J Ophthalmol. **1988;** 36(2): 98-99.

S99. Kobayashi T, Hayakawa K, Mawatari M, Itoh M, Akao N, Yotsu RR, *et al.* Loiasis in a Japanese traveler returning from Central Africa. Trop Med Health. **2015;** 43(2): 149-153.

S100. Gobbi F, Boussinesq M, Mascarello M, Angheben A, Gobbo M, Rossanese A, *et al.* Case report: loiasis with peripheral nerve involvement and spleen lesions. Am J Trop Med Hyg. **2011;** 84(5): 733-737.

S101. Gibbs RD. Loiasis: report of three cases and literature review. J Natl Med Assoc. **1979;** 71(9): 853-854.

S102. Peñafiel-Freire DM, Herranz-Aguirre M. Loiasis, a subconjunctival manifestation. J Pediatr. **2017;** 188: 300.

S103. Le Guyadec T, Wolkenstein P, Ortoli JC, Ponties-Leroux B, Beaulieu P, Millet P. [Foreign body granuloma on calcified loa loa filariasis]. Ann Dermatol Venereol. **1992;** 119(2): 127-130 (in French).

S104. Brady FJ, Ewing RE, Sanger S. Loaiasis: recovery of adult *Loa loa*. Ariz Med. **1963;** 20: 141-142.

S105. Vey EK. Filaria -- Loa-loa: case report. Ann Ophthalmol. **1975;** 7(3): 389-392.

S106. Chhabra RC, Bhat S, Shukla SM. Occular loiasis in a Zambian woman. East Afr Med J. **1989;** 66(7): 491-494.

S107. Jaffres R, Simitzis Le Flohic AM, Chastel C. [*Loa loa* filarial arthritis microfilaria in the joint fluid]. Rev Rhum Mal Osteoartic. **1983;** 50(2): 145-147 (in French).

S108. Zue N'Dong CHF. [Endocular filariasis apropos of a case of filaria *Loa loa* in the anterior chamber]. Bull Soc Ophtalmol Fr. **1985;** 85(2): 237-238 (in French).

S109. Stein MF Jr, Finkelstein WE. Loiasis in Westchester County. N Y State J Med. **1979;** 79(12): 1882-1883.

S110. Gasparri V, Cantera E, Guinetti C. Ocular filariasis from *Loa loa*: case report. Ital J Ophthalmol. **1993;** 7(3): 157-159.

S111. Fleury P, Liotet S, Carme B, Sainte-Laudy J, Haut J, Gentilini M. [Ocular loaiasis in a young Caucasian tourist]. J Fr Ophtalmol. **1980;** 3(8-9): 503-506 (in French).

S112. Ongom VL. *Loa loa* in Uganda (case report). East Afr Med J. **1974;** 51(3): 296-298.

S113. López-Rodríguez I, De-la-Fuente-Cid R, Carnero-López JM, Cordido-Carballido M, Zúñiga-Rodríguez C. [Loiasis. Approach to a form of ocular parasitosis]. Arch Soc Esp Oftalmol. **2007;** 82(1): 55-57 (in Spanish).

S114. Lortholary O, Jaccard A, Visser H, Guillevin L. [Prevention of human *Loa loa* filariasis]. Ann Med Interne (Paris). **1989;** 140(4): 319 (in French).

S115. Lucot J, Chovet M. [Intra-ocular loasis. Apropos of one case]. Rev Int Trach. **1973;** 50(4): 103-105.

S116. Malik STA, McHugh M, Morley AR, Ngu J, Qureshi M, Wilkinson R. Filariasis (*Loa loa*) associated with membranous glomerulonephritis. Kidney Int. **1981;** 20(1): 157 (Abstract, Meeting of the Society of Nephrology, German speaking. Würzburg, Germany, October 19-22, 1980).

S117. Britton CA, Sumkin J, Math M, Williams S. Mammographic appearance of loiasis. Am J Roentgenol. **1992;** 159(1): 51-52.

S118. Marriott WR. Loiasis in a young child in Oregon. Int J Dermatol. **1986;** 25(4): 252-254.

S119. Martin Jiménez ML, Fernández Tomás M, Molina Avila P, Montero Hernández E. [Intraocular foreign body after a trip to Cameroon]. Med Clin (Barc). **2016;** 147(11): e65 (in Spanish).

S120. Bouvet JP, Thérizol M, Auquier L. Microfilarial polyarthritis in a massive *Loa loa* infestation. A case report. Acta Trop. **1977;** 34(3): 281-284.

S121. Morrone A, Franco G, Toma L, Tchangmena OB, Marangi M. A case of loiasis in Rome. J Eur Acad Dermatol Venereol. **2002;** 16(3): 280-283.

S122. Nam JN, Reddy S, Charles NC. Surgical management of conjunctival loiasis. Ophthal Plast Reconstr Surg. **2008;** 24(4): 316-317.

S123. Nayak B, Sinha S, Nayak L. *Loa loa* in the vitreous cavity of the eye. BMJ Case Rep. **2016;** 2016. pii: bcr2015213879.

S124. Negesse Y, Lanoie LO, Neafie RC, Connor DH. Loiasis: "Calabar" swellings and involvement of deep organs. Am J Trop Med Hyg. **1985;** 34(3): 537-546.

S125. Tomaszunas S, Rozmarynowska M. Loaiasis in a visitor to Congo. Biul Inst Med Morsk Gdansk. **1967;** 18(1): 51-54.

S126. Nieves-Moreno M, Bañeros-Rojas P, Díaz-Valle D, Gegúndez-Fernández JA. [Encephalitis secondary to diethylcarbamazine treatment in a patient with ocular loiasis]. J Fr Ophtalmol. **2017;** 40(6): e229-e230 (in French).

S127. Hubler WR Jr, Gregory JF, Knox JM, Fall H. Loaiasis. A case report and review of literature. Arch Dermatol. **1973;** 108(6): 835-836.

S128. Roussel F, Roussel C, Brasseur P, Gourmelen O, Le Loet X. Aseptic knee effusion with *Loa loa* microfilariae in the articular fluid. Acta Cytol. **1989;** 33(2): 281-283.

S129. Giliani G, Chenal L, De Filippis M, Tosoni A, Corbellino M, Galimberti L, *et al. Loa loa* African eye worm: two cases in a non-endemic country, Italy. Clin Microbiol Infect. **2011;** 17(Suppl 4): S212 (Abstract, 21st European Congress of Clinical Microbiology & Infectious Diseases. Milan, Italy, May 7-10, 2011).

S130. Satyavani M, Rao KN. Live male adult *Loa loa* in the anterior chamber of the eye -- a case report. Indian J Pathol Microbiol. **1993;** 36(2): 154-157.

S131. de Brux JA, Baup HF, Kaeding H. *Loa loa* microfilariae in an endometrial smear. Acta Cytol. **1983;** 27(5): 547-549.

S132. Siganos S. Ocular loiasis in South Sudan. An Inst Barraquer, **1977;** 13(1-2): 67-70.

S133. Vermeer BJ, van der Kaay HJ. Loiasis: a case report. Acta Derm Venereol. **1982;** 62(1): 78-79.

S134. Oberg MS, McGowen BA, Kleiman DA. Loiasis 15 years after exposure. Tex Med. **1987;** 83(2): 36-37.

S135. Gerster JC, Favrat B, Buchard PA. [Articular involvement after a stay in Africa. Report of 3 cases of loiase]. Rhumatologie. **1994;** 46(6): 153-156 (in French).

S136. Carme B, Botaka E, Lehenaff YM. [Dead *Loa loa* filaria in a subconjunctival site. Apropos of a case]. J Fr Ophtalmol. **1988;** 11(12): 865-867 (in French).

S137. Bluestone G, Lariviere M, Ruskin J. *Loa loa*: a cause of chronic interstitial pneumonitis. Clin Infect Dis. **1997;** 25(2): 385 (Abstract, 35th Annual Meeting of the Infectious Diseases Society of America, San Francisco, California, USA, September 13-16, 1997).

S138. Wickremesinghe RS, Goonesinghe SK, Samarasinghe S. *Loa loa* in a Sri Lankan expatriate from Nigeria. Ceylon Med J. **1989;** 34(1): 31-34.

S139. Lee BY, McMillian R. *Loa loa*: ocular filariasis in an African student in Missouri. Ann Ophthalmol. **1984;** 16(5): 456-458.

S140. Novak R. Calcifications in the breast in filaria loa infection. Acta Radiol. **1989;** 30(5): 507-508.

S141. Ward TP, Laver NVM, Hidayat AA, Amacher AG III, Neafie RC, Simon DP, *et al.* A case of eyelid involvement in systemic loiasis. Clin Surg Ophthalmol. **2004;** 22(12): 374-376.

S142. Mandal D, Roy D, Bera DK, Manna B. Occurrence of gravid *Loa loa* in subconjunctival space of man: a case report from West Bengal, India. J Parasit Dis. **2013;** 37(1): 52-55.

S143. Burgués-Ceballos A, Marcos MA, March GA, Juberias JR. [Ocular loiasis in a patient with chronic hypereosinophilia]. Arch Soc Esp Oftalmol. **2014;** 89(10): 411-413 (in Spanish).

S144. Olness K, Franciosi RA, Johnson MM, Freedman DO. Loiasis in an expatriate American child: diagnostic and treatment difficulties. Pediatrics. **1987;** 80(6): 943-946.

S145. Osuntokun O, Olurin O. Filarial worm (*Loa loa*) in the anterior chamber. Report of two cases. Br J Ophthalmol. **1975;** 59(3): 166-167.

S146. Passos RM, Barbosa CP, Almeida J de S, Ogawa GM, Camargo LMA. Subconjunctival *Loa loa* worm: first case report in Brazil. Arq Bras Oftalmol. **2012;** 75(1): 67-70.

S147. Patel CK, Churchill D, Teimory M, Tabendeh H. Unexplained foreign body sensation: thinking of loiasis in at risk patients prevents significant morbidity. Eye (Lond). **1993;** 7(5): 714-715.

S148. Petersen S, Rønne-Rasmussen J, Basse P. Thrombosis of the ulnar veins - an unusual manifestation of *Loa loa* filariasis. Scand J Infect Dis. **1998;** 30(2): 204-205.

S149. Pillay VK, Kirch E, Kurtzman NA. Glomerulopathy associated with filarial loiasis. JAMA. **1973;** 225(2): 179.

S150. Le Lourd R, Vallet G, Dubourg P, Inquimbert P. [Radiographies of the extremities of a patient with *Loa loa*]. J Med Bord. **1961;** 138: 671 (in French).

S151. Rahal A, Marty P, Gari-Toussaint M, Le Fichoux Y, Castela J, Fuzibet JG. [Inflammatory arthropathy caused by *Loa loa* filariasis. A case]. Presse Med. **1992;** 21(38): 1824 (in French).

S152. Rakita RM, White AC Jr, Kielhofner MA. *Loa loa* infection as a cause of migratory angioedema: report of three cases from the Texas Medical Center. Clin Infect Dis. **1993;** 17(4): 691-694.

S153. O'Connell EM, Nutman TB. Reduction of *Loa loa* microfilaremia with imatinib - A case report. N Engl J Med. **2017;** 377(21): 2095-2096.

S154. Richardson ET, Luo R, Fink DL, Nutman TB, Geisse JK, Barry M. Transient facial swellings in a patient with a remote African travel history. J Travel Med. **2012;** 19(3): 183-185.

S155. Berenguer A, Plancha E, Muñoz Gil J. Right ventricular endomyocardial fibrosis and microfilarial infection. Int J Cardiol. **2003;** 87(2-3): 287-289.

S156. Rotaru L, Serban C. An extremely rare situation - subcutaneously filariasis presented at ED Craiova. Curr Health Sci J. **2014;** 40(2): 139-140.

S157. Ruben FL, Hines SL, Williams SL, Nathan G, Mendelow H. Loiasis in an American naturalist. Am J Trop Med Hyg. **1983;** 32(4): 738-740.

S158. Same Ekobo A, Same-Voisin C, Eben-Moussi E, Ongmagne MJ. A propos d'un cas de méningo-encéphalite filarienne à *Loa loa*. Rappels des critères de diagnostic de certitude. Afr Med. **1981;** 20(191): 359-361.

S159. Sarkany I. Loiasis with involvement of peripheral nerves. Trans St Johns Hosp Dermatol Soc. **1959;** 42: 49-51.

S160. Shah AN, Saldana M. Images in clinical medicine. Ocular loiasis. N Engl J Med. **2010;** 363(11): e16.

S161. Sirvent N, Thouret MC, Marty P, Boutte P, Mariani R. [The cost of a consultation...]. Arch Pediatr. **1999;** 6(9): 1027 (in French).

S162. Sreedharan S, Ullal S, Kamath MP, Hegde MC, Bhojwani KM, Alva A, *et al.* Solitary nasal mass: an unusual diagnosis. Am J Otolaryngol. **2011;** 32(4): 358-359.

S163. Sparrow CH, Goldsmid JM. Imported loiasis in Rhodesia. Cent Afr J Med. **1974;** 20(7): 143-146.

S164. Stelow EB, Pambuccian SE, Bardales RH, Cartwright CP, Reif CJ, Stanley MW. *Loa loa* presenting in a ThinPrep® Pap test^{TM}: case report and review of parasites in cervicovaginal cytology specimens. Diagn Cytopathol. **2003;** 29(3): 167-171.

S165. Khetan VD. Subconjunctival *Loa loa* with calabar swelling. Indian J Ophthalmol. **2007;** 55(2): 165-166.

S166. Suneja SK, Grigsby ME, Olopoenia L, Debruhl N, Teal JS. Mammographic calcifications due to filarial disease. Trop Doct. **1990;** 20(3):143-144.

S167. Taiwo SS, Tamiowo MO. *Loa loa* meningoencephalitis in Southwestern Nigéria. West Afr J Med. **2007;** 26(2): 156-159.

S168. Varenne F, Fillaux J, Porterie M, Soler J, Cassagne M, Soler V. [Subconjunctival loiasis: A case report]. J Fr Ophtalmol. **2016;** 39(8): e193-e194 (in French).

S169. Vedy J, Cahuzac G, Labegorre J. Manifestations oculaires atypiques des filarioses à Loa-Loa. Méd Armées. **1975;** 3(9): 739-746.

S170. Fenton P. *Loa loa*: the African eye worm. Arch Ophthalmol. **1966;** 76(6): 866-867.

S171. Sachs HG, Heep M, Gabel VP. [Surgical worm extraction in *Loa loa* ophthalmia]. Klin Monbl Augenheilkd. **1998;** 213(6): 367-369 (in German).

S172. Vandellen RG, Ottesen EA, Gocke TM, Neafie RC. *Loa loa* - an unusual case of chronic urticaria and angioedema in the United States. JAMA. **1985;** 253(13): 1924-1925.

S173. Pogemiller H, Settgast A. *Loa loa* in a Congolese refugee woman in Minnesota. Am J Trop Med Hyg, **2014;** 91(5 Suppl): 148 (Abstract, 63rd Annual Meeting, American Society of Tropical Medicine and Hygiene, New Orleans, Louisiana, USA, November 2-6, 2014).

S174. Doury P, Saliou P, Charmot G. [Articular effusions with eosinophils. Apropos of a case report]. Sem Hop. **1983;** 59(22): 1683-1685 (in French).

S175. Harley RD. *Filaria loa*: report of a case. Am J Ophthalmol. **1958;** 45(6): 901-904.

S176. Dos Santos J, Irby R, Allen G. Calabar swelling in Virginia. Va Med Mon. **1965;** 92: 226-228.

S177. Rivoire J, Castel J, Despeignes J, Battesti MR. [A case of *Loa loa* filariasis]. Lyon Med. **1965;** 214(33): 211-212 (in French).

S178. Bendelac J. [*Loa loa* filariasis: 2 cases observed at the Hôpital de l'O.C.P. (Khouribga)]. Maroc Med. **1971;** 51(544): 174-175 (in French).

S179. Ellias SF, Dimanin JV, Kaftan SN, Phillips TH. *Loa Loa*: Report of case of infection. J Am Osteopath Assoc. **1964;** 63: 646-649.

S180. Geldelman D, Blumberg R, Sadun A. Ocular Loa Loa with cryoprobe extraction of subconjunctival worm. Ophthalmology. **1984;** 91(3): 300-303.

S181. Garnier C, Dujardin P, Campagni JP, Simony J, Lefichoux Y. [Two observations of *Loa loa* filariasis. Value of the diethylcarbamazine test]. Sem Hop. **1981;** 57(37-38): 1535-1536 (in French).

S182. Renard G, Morand L, Lacombe E, Offret G. [A case of retinal filariasis]. J Fr Ophtalmol. **1978;** 1(1): 41-46 (in French).

S183. Wilms G, Tschibwabwa-Ntumba E, Nijssens M, Baert AL. Calcified loa-loa infestations. J Belge Radiol. **1983;** 66(2): 133-136.

S184. Berthou JD, Garin JP, Loire R, Rassat JP, Jean R, Jocteur-Monrozier D, *et al.* [Fatal endomyocardial fibrosis with massive embolism in a case of filariasis: discussion of filarial cardiopathy and therapeutic problems]. Lyon Méd. **1973;** 229(9): 903-912 (in French).

S185. Fleck BW, Macdonald M. Periocular *Loa loa* in eastern Scotland: a report of two cases. J R Coll Surg Edinb. **1987;** 32(3): 163.

S186. Morel L, Delaude A, Girard M, Bouzekri M. [Eosinophilic pulmonary infiltrates in filariasis of the *Loa loa* type]. Poumon Coeur. **1967;** 23(6): 685-694 (in French).

S187. Dufour D, Francois P, Madelain F. [Endocular filariasis]. Bull Soc Ophtalmol Fr. **1973;** 73(3): 491-494 (in French).

S188. Campo S, Carta S, Gasparri V, Nowakowski M. Peripheral eosinophilia due to '*Loa loa*' infection. Eur J Intern Med. **1995;** 6(2): 127-128.

S189. Portilla Sogorb J, Sevilla Llinares A, Carrión A, Cordóba C, Blesa M. [Leukocytoclastic vasculitis due to hypersensitivity to microfilariae]. An Med Interna (Madrid). **1991;** 8(1): 30-32 (in Spanish).

S190. Pedro-Egbe CN, Chukwuka IO, Obunge OK. Live adult Loa-Loa in the anterior chamber of a Nigerian female. Port Harcourt Med J. **2008;** 3(1): 104-107.

S191. Legrand J, Baron A, Hervouet F, Chevannes H, Ginguene A. [Apropos of 2 Cases of *Loa loa* Filariasis]. Bull Soc Ophtalmol Fr. **1964;** 64: 341-344 (in French).

S192. Gentilini M, Domart A, Brumpt L, Hazard J, Lequintrec Y. [*Loa loa* filariasis and proteinuria]. Bull Soc Pathol Exot Filiales. **1963;** 56: 207-217 (in French).

S193. Michotte L, De Schrevel J. [*Loa loa* filariasis and the rheumatoid factor]. J Belge Med Phys Rhumatol. **1962;** 17: 241-243 (in French).

S194. Becquet R. [Filariasis caused by *Loa loa*]. J Sci Med Lille. **1960;** 78: 227-238 (in French).

S195. Casquero Murciego A, Rivas González P, de Górgolas Hernández-Mora M. [39 year old woman with eosinophilia and subcutaneous edema after stay in tropical areas]. Rev Clin Esp. **2005;** 205(11): 571-572 (in Spanish).

S196. Franco-Alvarez de Luna F, Giménez-Almenara G, Vidal E, Casal M. [Patient from the Republic of Congo with intermittent ocular pain]. Enferm Infecc Microbiol Clin. **2007;** 25(3): 215-216 (in Spanish).

S197. Gálvez-López R, Chueca-Porcuna N, Fuertes-Rodríguez A, Muñoz-Medina L. [Loiasis, a cause of eosinophilia to consider]. Rev Clin Esp (Barc). **2013;** 213(9): e99 (in Spanish).

S198. Vivas Moresco M, García Lopez Hortelano M, Villota Arrieta J, Subirats Núñez M. [Guinean child with Calabar swelling: loiasis]. An Pediatr (Barc). **2017;** 86(6): 359-360 (in Spanish).

S199. Puente S, Moneo I, Subirats M, Martínez M, Lago M, González-Lahoz JM. [Filariasis caused by *Loa loa*]. Enferm Infecc Microbiol Clin. **1994;** 12(4): 227 (in Spanish).

S200. Saldarreaga A, García-Gil D, Soto-Cárdenas MJ, García-Tapia AM. [Recurrent angioedema in a young woman]. Enferm Infecc Microbiol Clin. 2004; 22(7): 430-431 (in Spanish).

S201. Pérez Pérez AJ, Sobrado J, Cigarrán S, Valdés R, González L, Courel M. [End-stage renal disease due to filarial loiasis]. Nefrología. **1987;** 7(2): 183-185 (in Spanish).

S202. Altozano F. [Filariasis loa in a 3-year-old child]. Acta Pediatr Esp. **1962;** 20: 247-252 (in Spanish).

S203. Moreno Martínez MP, Marzo Gracia J, Marrón Moya SE, Marrón Gasca J. [A case of loaiasis]. Actas Dermosifiliogr. **1990;** 81(7-8): 501-502 (in Spanish).

S204. Grasa Jordán MP, Marrón Gasca J, Gómez Lus R, Rubio Calvo C, Navarro Lucia M. [Filariasis caused by *Loa loa* (loaiasis)]. Actas Dermosifiliogr. **1979;** 70(7-8): 467-474 (in Spanish).

S205. Sanchís-Bayarri Vaillant V, Bataller Blaco H, Fraile MT. [Loa-Loa filariasis associated with thrombophlebitis]. Rev Diagnost Biol. 1987; 36(1): 54 (in Spanish).

S206. Cuadros J, Romanyk J, González-Palacios R, Gómez-Herruz P, Beltrán M. [Ocular sensation of foreign body and generalized pruritus in a young Guinean woman]. Enferm Infecc Microbiol Clin. **1998;** 16(1): 39-41 (in Spanish).

S207. Sanchís-Bayarri Vaillant V, Lorente Ortuno S, Bataller Blasco H. [Hypereosinophilia caused by loa-loa filaria]. Med Clin (Barc). **1984;** 82(18): 825 (in Spanish).

S208. de la Herrán Herrera A, González Garrido EA, Pila Pérez R, León Díaz R. [Myocardiopathy in a patient with loaiasis. Presentation of a case]. Rev Cubana Med Trop. **1981;** 33(3): 201-206 (in Spanish).

S209. Thomas J, Chastel C, Forcain L. [Clinical and parasitic latency in filariasis due to *Loa loa* and *Onchocerca volvulus*]. Bull Soc Pathol Exot Filiales. **1970;** 63(1): 90-94 (in French).

S210. Cherel P, Ouhioun O, Hagay C, De Maulmont C. [Breast filariasis: report of two cases]. Sein. **1998;** 8(4): 237-254 (in French).

S211. Popescu MP, Paun L, Panaitescu D. [Neuro-chorio-retinal manifestations in *Onchocerca volvulus* and *Loa loa* filariasis associated with malaria]. Bull Mem Soc Fr Ophtalmol. **1986;** 97: 54-56 (in French).

S212. Crosnier R, Darbon A, Dulac JF, Quilicchini F. [Filariosis loa and amebiasias and their therapy with notezine]. Bull Soc Pathol Exot Filiales. **1953;** 46(5): 702-708 (in French).

S213. Heckenroth F, Becuwe R, Mayan L, Leroux G. Filarioses (*loa* et *perstans*) et dérivés de la pipérazine. Bull Soc Pathol Exot Filiales. **1950;** 43(5-6): 354-363.

S214. Gomez Lus R. [*Wuchereria bancrofti* and *Loa loa*: a case of loaiasis]. Med Trop (Madr). **1965;** 41(5): 408-417 (in Spanish).

S215. Lagrange E. [Treatment of filariases due to *Loa loa* and *O. volvulus* by diethylcarbamazine chloride]. Ann Soc Belg Med Trop. **1949;** 24(1): 19-22 (in French).

S216. Vuillemin J, Stines J, Gilles R. From Cameroon to Luxemburg, a mammary immigration of filariae. Sein. **2000;** 10(3):157-162.

S217. Pannier R, Janssens PG, Verstraeten J, Van Genabeek A. [Contribution to the study of filarial cardiopathy]. Mal Cardiovasc. **1964;** 5(1): 113-116 (in Italian).

S218. Van Hoegaerden M, Flocard F. Treatment of recurrent, filarial, Calabar-type oedema with mebendazole. Trop Geogr Med. **1986;** 38(3): 296-298.

S219. Orlando G, Galli M, Lazzarin A, Serino G, Inzoli C, Calello G, *et al*. [Humoral immune responses in human loaiasis]. Boll Ist Sieroter Milan. **1982;** 61(3): 258-261 (in Italian).

S220. Ekwere PD. Filarial orchitis: a cause of male infertility in the tropics--case report from Nigeria. Cent Afr J Med. **1989;** 35(8): 456-460.

S221. León D, Martín M, Corros C, Santamarta E, Costilla S, Lambert JL. Usefulness of cardiac MRI in the early diagnosis of endomyocardial fibrosis. Rev Port Cardiol. **2012;** 31(5): 401-402.

S222. Charles MK, Mansour S, Tomlin P, Kowalewska-Grochowska K. That feeling of being out of place: a microfilarial tale. Am J Trop Med Hyg. 2016; 95(5 Suppl): 154. (Abstract, 65th Annual Meeting, American Society of Tropical Medicine and Hygiene, Atlanta, Georgia, USA, November 13-17, 2016).

S223. Lukiana T1, Mandina M, Situakibanza NH, Mbula MM, Lepira BF, Odio WT, *et al.* A possible case of spontaneous *Loa loa* encephalopathy associated with a glomerulopathy. Filaria J. **2006;** 5: 6.

S224. Abel L, Joly V, Yeni P, Carbon C, Bussel A. Apheresis in the management of loiasis with high microfilariaemia and renal disease. Br Med J (Clin Res Ed). **1986;** 292(6512): 24.

S225. Boussinesq M, Bain O, Chabaud AG, Gardon-Wendel N, Kamgno J, Chippaux JP. A new zoonosis of the cerebrospinal fluid of man probably caused by *Meningonema peruzzii*, a filaria of the central nervous system of Cercopithecidae. Parasite. **1995;** 2(2): 173-176.

S226. Chang LW, Reller ME, Bishop JA, Talaat K, Nutman TB, Auwaerter PG. A 41-year-old woman from Cameroon with infertility. Clin Infect Dis. **2008;** 47(1): 141-143, 109.

S227. Arcos Pereda P, Rojo Marcos G, Velo Plaza M, Cuadros González J, García García E. Complete remission of loiasis-associated nephrotic syndrome with collapsing glomerulopathy after diethilcarbamazine treatment. Enf Emerg. **2010;** 12(3): 155-158.

S228. Klion AD, Eisenstein EM, Smirniotopoulos TT, Neumann MP, Nutman TB. Pulmonary involvement in loiasis. Am Rev Respir Dis. **1992;** 145(4, Pt 1) :961-963.

S229. Landry P, Bassi C, Christen B. *Loa loa* infection in a patient with thymoma. Travel Med Infect Dis. **2004;** 2(2): 85-87.

S230. McCallister S, Flaherty JP. Loiasis in a Peace Corps volunteer from Zaire. Clin Microbiol Newsl. **1993;** 15(2): 12-16.

S231. Priest DH, Nutman TB. Loiasis in US traveler returning from Bioko Island, Equatorial Guinea, 2016. Emerg Infect Dis. **2017;** 23(1): 160-162.

S232. Nutman TB, Kradin RL. Case records of the Massachusetts General Hospital. Weekly clinicopathological exercises. Case 1-2002. A 24-year-old woman with paresthesias and muscle cramps after a stay in Africa. N Engl J Med. **2002;** 346(2): 115-122.

S233. Scott JAG, Davidson RN, Moody AH, Bryceson ADM. Diagnosing multiple parasitic infections; trypanosomiasis, loiasis and schistosomiasis in a single case. Scand J Infect Dis. **1991;** 23(6): 777-780.

S234. Pakasa NM, Nseka NM, Nyimi LM. Secondary collapsing glomerulopathy associated with *Loa loa* filariasis. Am J Kidney Dis. **1997;** 30(6): 836-839.

S235. De Decker W. [Extraction of *Loa loa* filaria from the subconjunctival space]. Ber Zusammenkunft Dtsch Ophthalmol Ges. **1970;** 70: 96-99 [in German].

S236. Grupp A. [A case of loa-loa filariasis]. Klin Monbl Augenheilkd. **1975;** 167(1): 70-76 (in German).

S237. Hagen H. [A case of *Loa loa*]. Med Welt. **1973;** 24(18): 751-752 (in German).

S238. Hartwig H, Sachsenweger R. [Recent problems in ophthalmologic diagnostics. Surgical removal of a loaloa filaria from the conjunctiva of a negro]. Wiss Z Karl Marx Univ Math Naturwiss. **1964;** 13(2): 213-214 (in German).

S239. Hess C. [A case from general practice. Filariasis due to *Loa loa*]. Schweiz Rundsch Med Prax. **1985;** 74(20): 541-542 (in German).

S240. Jaksche A, Wessels L, Martin S, Loeffler KU. [Ocular involvement in systemic Loa-Loa filariasis. Case report and review of the literature]. Ophthalmologe. **2004;** 101(9): 931-934 (in German).

S241. Kager PA. [Eosinophilia; a worm infection from the tropics]. Ned Tijdschr Geneeskd. **1989;** 133(44): 2167-2170 (in Dutch).

S242. Krautheim H. [Loaiasis. Clinical aspects and therapy in the field of dermatology]. Med Welt. **1982;** 33(23): 837-839 (in German).

S243. Lange CE. [Symptomatology and therapy of loaiasis]. Aktuelle Derm. **1977;** 3(2): 165-170 (in German).

S244. Prüfer L. [Differential diagnosis at the bedside. Itching skin edemas - allergy or parasitosis?]. Munch Med Wochenschr. **1983;** 125(16): Suppl 105 (in German).

S245. Rudigier J, Schneider HM, Paulini K, Rückert K. [Chronic tendovaginitis in parasitic diseases]. Chirurg. **1978;** 49(12): 769-775 (in German).

S246. Schouten SB, Vukadin M, Naafs B, Visser LG. [Forearm swelling and eosinophilia; the importance of travel anamnesis]. Tijdschr Infect. **2010;** 5(3): 110-114 (in Dutch).

S247. Stähelin AG1, Nüesch R. [Patient from Cameroon with recurrent, transient, itching swelling of arms and legs]. Praxis (Bern). **2006;** 95(37): 1423-1425 (in German).

S248. Stammen J, Klüppel M. [Bilateral subconjunctival foreign bodies. Loiasis with bilateral *Loa* ophthalmia]. Ophthalmologe. **2002;** 99(4): 304-305 (in German).

S249. Stemmle J, Markwalder KA, Zinkernagel AS, Wirth MC, Grimm F, Hirsch-Hoffmann S, *et al.* [*Loa loa* infection of the eye -- a case series]. Klin Monbl Augenheilkd. **2005;** 222(3): 226-230 (in German).

S250. Uhlig CE, Dietrich M, Busse H. [Asthenoscopic problems with a subconjunctival worm]. Ophthalmologe. **2002;** 99(11): 880-881 (in German).

S251. Van Kortenhof NMM, Jaspers CAJJ, Sanders CJG. [A patient with loiasis following a trip to Central Africa]. Ned Tijdschr Geneeskd. **2003;** 147(27): 1327-1329 (in Dutch).

S252. Werner H. [Demonstration of subconjunctival *Filaria loa* with an 8 mm. film recording]. Ophthalmologica. **1959;** 137(3): 160-162 (in German).

S253. Kroon BBR. [*Loa loa*: the 'eyeworm']. Ned Tijdschr Geneeskd. **1981;** 125(5): 199-200 (in Dutch).

S254. Sebus J, Mouton RP. [A patient with Loa-Loa filariasis]. Ned Tijdschr Geneeskd. **1970;** 114(52): 2193-2194 (in Dutch).

S255. Van Kerckhove H. [Filarial rheumatism]. Belg Tijdschr Geneeskd. **1960;** 16(23): 1156-1162 (in Dutch).

S256. Kamgno J, Boussinesq M, Labrousse F, Nkegoum B, Thylefors BI, Mackenzie CD. Case report: encephalopathy after ivermectin treatment in a patient infected with *Loa loa* and *Plasmodium* spp. Am J Trop Med Hyg. **2008;** 78(4): 546-551.

S257. Cauchie C, Rutsaert J, Thys O, Bonnyns M, Perier O. [Encephalitis due to Loa-Loa, treated with the combination of cortisone and carbamazine]. Rev Belg Pathol Med Exp. **1965;** 31(3): 232-244 (in French).

S258. Van Bogaert L, Dubois A, Janssens PG, Radermecker J, Tverdy G, Wanson M. Encephalitis in loa-loa filariasis. J Neurol Neurosurg Psychiatry. **1955;** 18(2): 103-119.

S259. Nzenze JR, Kombila MY, Boguikouma JB, Belembaogo E, Moussavou-Kombila JB, Nguemby-Mbina C. Encéphalopathie mortelle au cours d'une loase hypermicrofilaremique

traitee par ivermectine: première description au Gabon. Med Afr Noire. **2001;** 48(8/9): 375-377.

S260. Cattan R, Frumusan P, Levy C. [Filarial encephalopathy]. C R Hebd Seances Acad Sci. **1960;** 76(21-22): 808-812 (in French).

S261. Kivits M. [Four cases of fatal encephalitis with invasion of the cerebrospinal fluid by Microfilaria loa]. Ann Soc Belg Med Trop. **1952;** 32(3): 235-242 (in French).

S262. Garin C, Garin JP. [Treatment of filariasis from *Filaria loa* by notezine]. J Med Bord. **1951;** 128(1): 250-252 (in French).

S263. Brumpt LC, Pequignot H, Lhermitte F, Petithory J, Remy H. [Therapeutic encephalitis by loiasis with a high microfilaremia; treatment by blood exchange transfusion]. Bull Mem Soc Med Hop Paris. **1966;** 117(11): 1049-1058 (in French).

S264. Kuhlencord A, Bommer W, Nitsche R, Lamberts R. [Successful therapy of filarial infections (*Loa loa*, *Mansonella perstans*) with mebendazole]. Mitt Österr Ges Tropenmed Parasitol. **1990;** 12: 171-177 (in German).

S265. Clausen M, Roider J, Fuhrmann C, Laqua H. [Stabbing pain, conjunctival changes, foreign body sensation and unilateral red eye. Subconjunctival macrofilariasis in systemic *Loa loa* filariasis]. Ophthalmologe. **1998;** 95(1): 56-57 (in German).

S266. Toussaint D, Danis P. Retinopathy in generalized loa-loa filariasis. A clinicopathological study. Arch Ophthalmol. **1965;** 74(4): 470-476.

S267. Kenney M, Hewitt R. Psycho-neurotic disturbances in filariasis, and their relief by removal of adult worms or treatment with hetrazan. Am J Trop Med. **1950;** 30(6): 895-899.

S268. Arrey-Agbor DB, Nana-Djeunga HC, Mogoung-Wafo AE, Mafo M, Danwe C, Kamgno J. Case report: Probable case of spontaneous encephalopathy due to loiasis and dramatic reduction of *Loa loa* microfilariaemia with prolonged repeated courses of albendazole. Am J Trop Med Hyg. **2018;** 99(1): 112-115.

S269. Herrero-Morín JD, Fernández González MN, González Rodríguez F, García López E, Díaz Argüelles M. [Ocular filariasis due to Loa-loa. An emerging tropical parasitosis in Europe?]. An Pediatr (Barc). **2006;** 65(2): 168-170 (in Spanish).

S270. Sacks HN, Williams DN, Eifrig DE. Loiasis. Report of a case and review of the literature. Arch Intern Med. **1976;** 136(8): 914-915.

S271. Begle HL. Infestation with *Filaria loa*: Report of a case of filaria beneath the conjunctiva and microfilariae in the peripheral blood stream. J Am Med Assoc. **1921;** 76(19): 1301-1305.

S272. Pays JF, Ecalle JC, Cornet A, Brumpt L. [Neuropsychic manifestations of loaiasis. A clinical case of potomania]. Bull Soc Pathol Exot Filiales. **1976;** 69(3): 265-272 (in French).

S273. Bada JL, Fernández-Nogues F, Cerda E, Rufi G. Neurological manifestations in a patient with filariasis. Br Med J. **1976;** 2(6042): 978-979.

S274. Zuidema PJ. [Loiasis]. Ned Tijdschr Geneeskd. **1966;** 110(4): 165-167 (in Dutch).

S275. Malouf AR, Marty AM, Vaxmonsky TP. Loiasis in Maryland. Arch Ophthalmol. **1992;** 110(7): 1010.

S276. Mayer J. [Contribution on filariasis Loa-Loa]. Wien Med Wochenschr. **1966;** 116(14): 296-299 (in German).

S277. Verallo O, Fragiotta S, Carnevale C, De Rosa V, Vingolo EM. [Subconjunctival loiasis: case reports and review of cases described in Italy]. Clin Ter. **2013;** 164(2): e127-e131 (in Italian).

S278. Sgrelli A, De Socio GV, Papili R, D'Annibale ML, Baldelli F. [*Loa loa* filariasis in Italy: review of the literature with a clinical report]. Infez Med. **2011;** 19(3): 147-151 (in Italian).

S279. Gil-Setas A, Pérez Salazar M, Navascués A, Rodríguez Eleta F, Cebamanos JA, Rubio MT. [*Loa loa* and *Mansonella perstans* coinfection in a patient from Guinea]. An Sist Sanit Navar. **2010;** 33(2): 227-231 (in Spanish).

2. Atypical Ocular Manifestations

From the 32 patients with atypical ocular symptoms, there was no report of co-infections with other agents causing ocular manifestations such as *Onchocerca volvulus* and/or *Chlamydia trachomatis*, nor were onchocerciasis or trachoma mentioned as possible causes of the manifestations observed. Supplementary Table 1 summarizes the information on ocular manifestations; the references are those in Section 1: Literature Review (Supplementary References).

3. Eosinophilia and Co-Infections

There were 199 cases with eosinophilia (defined as a peripheral eosinophil blood count >0.5 x 10^9 cells or a peripheral blood eosinophil count ≥6% [1]. Of these, 182 (91.5%) did not have co-infecting helminths reported, but malaria was recorded at the time of consultation in 5, amoebiasis in 4 (one of whom also had *Trichuris trichiura*, one had hookworm, and one had *Strongyloides stercoralis*), trypanosomiasis in 1 (who also had schistosomiasis) and tuberculosis in 1. Most infectious causes of peripheral blood eosinophilia are parasitic, and particularly helminth infection; protozoa, in general, do not cause eosinophilia [2]. There were 17 (8.5%) cases with co-infecting helminths, 8 with *Mansonella perstans*, 1 with *O. volvulus*, 2 with *Ascaris lumbricoides*, 1 with *Trichuris trichiura*, 1 with hookworm, 1 with hookworm and *Ascaris*, 1 with *Strongyloides stercoralis*, and 2 with *Schistosoma* spp. (The case with *Loa loa* and *O. volvulus* did not have atypical ocular manifestations.)

Supplementary Table 1: Characteristics of Ocular Manifestations in the 32 Patients Identified with Loiasis Atypical Ocular Symptoms

Suppl. Ref.	Sex/Age (years or months)	Ocular Symptoms	Clinical Examination	Diagnosis of *Loa loa* and microfilarial density in blood (mf/ml) when reported	Ocular Symptoms After Treatment or Surgery
S26	M/26 yr	Unilateral loss of vision	Retinal edema, hemorrhages and retinal artery occlusion	*L. loa* microfilariae in blood (unspecified mf/ml)	Not reported
S42	F/60 yr	Unilateral loss of vision, ocular pain	*Loa loa* adult worm visualized in the anterior chamber	*L. loa* adult worm extraction from the anterior chamber	Not reported
S73	F/40 yr	Bilateral reduction in vision	Retinal hemorrhages and exudates, hemorrhages in the vitreous humour	*L. loa* microfilariae in blood (20,000 mf/ml)	Treated with DEC (diethylcarbamazine citrate) and had improvement in ocular symptoms
S78	F/25 yr	Unilateral loss of vision, ocular pain, erythema, photophobia, lacrimation, pruritus, foreign body sensation	*L. loa* adult worm visualized in the anterior chamber, conjunctival hyperemia	*L. loa* adult worm extraction from the anterior chamber	Treatment with albendazole and had progression to full loss of vision in the left eye
S93	M/48 yr	Unilateral blurring of vision, ocular pain, erythema, foreign body sensation, lacrimation, pruritus	*L. loa* adult worm visualized in the anterior chamber, conjunctival hyperemia	*L. loa* adult worm extraction from the anterior chamber	Not reported

S95	F/32 yr	Foreign body sensation, threadlike swelling of the left upper eyelid	Threadlike swelling in the left upper eyelid	*L. loa* adult worm extraction	Not reported
S108	Not reported/7 mo.	Not available due to patient age	Corneal edema, eye was firm to palpation	*L. loa* adult worm extraction from the anterior chamber	48 hr after extraction, the eye was no longer firm to palpation
S115	F/14 mo.	Photophobia, lacrimation	Corneal inflammation and swelling	*L. loa* adult worm extraction	Not reported
S123	M/25 yr	Unilateral reduction in vision with floaters, ocular pain, erythema	Grade one vitreous haze and corneal edema	*L. loa* adult worm extraction from the vitreous humour	Treated with DEC and had improvement in vision, the vitreous had cleared, minimal corneal edema
S130	M/11 yr	Erythema, photophobia, unilateral lacrimation	*L. loa* adult worm visualized in the anterior chamber	*L. loa* adult worm extraction from the anterior chamber	Not reported
S145	F/15 yr	Unilateral reduction in vision, ocular pain, foreign body sensation	*L. loa* adult worm visualized in the anterior chamber, conjunctival injection, hazy cornea	*L. loa* adult worm extraction from the anterior chamber	Eye was surgically removed and pathological examination showed a thick post inflammatory membrane adherent to the iris, hazy anterior chamber, choroidal and retinal detachment

S145	F/25 yr	Unilateral ocular pain, pruritus, foreign body sensation	*L. loa* adult worm visualized in the anterior chamber, discrete opacities in the cornea	*L. loa* adult worm extraction from the anterior chamber	Eye was surgically removed and pathological examination showed a fibrous membrane lining the anterior iris and lens, edematous ciliary body and iris, subcapsular fibrosis
S169	F/15 yr	Unilateral reduction in vision in the left eye	*L. loa* adult worm extending from anterior vitreous chamber to posterior chamber, total retinal detachment leading to rapid opacification of lens	*L. loa* adult worm extraction from the vitreous humour; *L. loa* microfilariae in blood (2,000 mf/ml)	Not reported
S169	F/19 yr	Unilateral reduction in vision	Live microfilariae in the vitreous humour with opacification of the vitreous humour	*L. loa* microfilariae in blood (unspecified mf/ml) and microfilariae from the vitreous cavity	Treatment with DEC decreased microfilarial density
S169	F/21 yr	Unilateral reduction in vision	Microfilariae in the vitreous cavity, panuveitis	*L. loa* microfilariae in blood (300,000 mf/ml) and microfilariae from the vitreous cavity	Not reported
S169	M/22 yr	Unilateral reduction in vision	Macular choroido-retinitis	*L. loa* microfilariae in blood (3,000 mf/ml)	Treatment with DEC and had regression of the retinal lesions with improvement in visual acuity

S169	M/25 yr	Bilateral reduction in vision	Retinal exudates and hemorrhages	*L. loa* microfilariae in blood (20,000 mf/ml)	Treatment with DEC and had a large hemorrhage in the vitreous cavity
S169	M/28 yr	Bilateral reduction in vision	Retinal exudates and hemorrhages, vein thrombosis	*L. loa* microfilariae in blood (50,000 mf/ml)	Treated with DEC and had progressive regression of the retinal lesions without improvement in vision
S169	F/30 yr	Bilateral reduction in vision	Inflammation of the vitreous humour, floating retinal detachments	*L. loa* microfilariae in blood (66,000 mf/ml)	Treatment with DEC and had development of vitreous fibrosis
S169	M/35 yr	Bilateral reduction in vision	Dead microfilariae in the vitreous, grey vitreous humour, opacified lens	*L. loa* microfilariae in blood (unspecified mf/ml) and microfilariae from the vitreous cavity	Treatment with DEC, development of a fibrosis of the vitreous, vision never recovered
S169	F/36 yr	Unilateral reduction in vision	Macular choroido-retinitis	*L. loa* microfilariae in blood (6,000 mf/ml)	Treatment with DEC and had regression of choroido-retinitis
S169	F/40 yr	Bilateral reduction in vision	Irido-cyclitis	*L. loa* microfilariae in blood (15,000 mf/ml)	Treatment with DEC and had resolution of the irido-cyclitis with complete recovery of vision
S169	M/46 yr	Unilateral reduction in vision	Dead microfilariae in the vitreous humour of the right eye, the right vitreous humour appeared grey, left lens opacification	*L. loa* microfilariae in blood (5,000 mf/ml) and microfilariae from the vitreous cavity	Treated with DEC but vision never recovered

S169	M/67 yr	Bilateral reduction in vision	Retinal exudates and hemorrhages, light opacification of lens	*L. loa* microfilariae in blood (10,000 mf/ml)	Treatment with DEC and had resolution of the retinal lesions with improvement in visual acuity
S182	M/12 yr	Bilateral loss of vision	Bilateral retinal haemorrhages with numerous exudates in peripheries, narrowing of arteries, neovascularisation	*L. loa* microfilariae in blood (4,500 mf/ml)	Treated with DEC and had resolution of haemorrhages and neovascularisation, regression of size of exudates with improvement in visual acuity
S187	F/24 yr	Unilateral reduction in vision	Macular degeneration, retinal detachment, peripheral hemorraghes, neovascularisation, scarring. Worm visualized in the sub-conjunctiva	*L. loa* adult worm extraction; *L. loa* microfilariae in blood (unspecified mf/ml)	Underwent surgery for retinal detachment without improvement in vision
S190	F/23 yr	Unilateral (left eye) reduction in vision, decreased visual acuity, ocular pain, pruritus, erythema	Panuveitis, elevated unilateral ocular pressure, hazy cornea. *L. loa* adult worm visualized in the anterior chamber	*L. loa* adult worm extraction from the anterior chamber	Treatment with DEC, topical and systemic steroids, without improvement in vision
S211	M/24 yr	Unilateral loss of vision	Retinal haemorrhages, retinal edema, pseudocystic opacification adjacent to macula	*L. loa* microfilariae in blood (unspecified mf/ml)	Treatment with DEC and had improvement in vision

S236	M/not reported	Blurring of vision and decreased visual acuity	Edema near the optic nerve	*L. loa* adult worm extraction from the conjunctiva	Treatment with DEC and had development of retinal edema that responded to corticosteroids
S250	M/30 yr	Ocular pain, foreign body sensation	Adult *L. loa* worm in the conjunctiva (failed extraction) with a lesion at level of macula to suggest passage of the worm	*L. loa* microfilariae in blood (unspecified mf/ml)	Not reported
S259	M/23 yr	Reduction in vision, pruritus	Anterior uveitis, microfilariae in the anterior chamber	*L. loa* microfilariae in blood (120,000 mf/ml) and microfilariae in the anterior chamber	Not reported
S266	M/38 yr	Bilateral reduction in vision, photophobia	Adult *L. loa* worm in the left upper eyelid, retinal hemorrhages and exudates, obstructed retinal vessels	Microfilariae in the Cerebrospinal fluid (CSF) and positive skin snip* (not due to onchocerciasis)	Not reported

* *Loa loa* microfilariae can be found in bloodless skin snips [3].

4. Statistical Analyses

4.1 Chi-Squared Test

Pearson's chi-squared test was used to test whether there was a statistically significant association between blood microfilarial density (MFD) levels and atypical presentation; where MFD levels had been categorised as: Zero (amicrofilaraemic, no mf detectable in blood smear), Low (greater than 0 but < 8,000 mf/ml) and High (≥ 8,000 mf/ml). All individuals for whom information on manifestation (either typical or atypical) and MFD were included. There are, therefore, more individuals included in this test than in the subsequent regression-based analyses, where data on a wider range of factors, specifically an individual's age, sex, residency status and MFD level (and eosinophilia status for Model 2) were required for them to be included.

Supplementary Table 2: Distribution of Individual Patients According to Presentation of Manifestations and Blood Microfilarial Density (MFD) Levels

	MFD Levels			
**Manifestations**	**Zero**	**Low**	**High**	**Total**
Typical	62	30	3	95
Atypical	40	21	16	77
Total	102	51	19	172

Pearson's chi-squared test using these data gave a chi-squared value of 13.492, which, with 2 degrees of freedom, yielded a p-value of 0.001176.

4.2 Multivariate Logistic Regression

Model 1: Atypical Presentation ~ Age + Sex + Residency Status + MFD Level (n = 154 individuals)

Model 2: Atypical Presentation ~ Age + Sex + Residency Status + MFD Level + Eosinophilia Status (n = 114 individuals)

We defined two multivariate logistic regression models (Model 1 and Model 2, the results of which are detailed in Figure 3 of the main text) that were fitted to the IPD collated through the systematic literature review. For both models, age was included as a continuous variable. Sex was defined as a categorical variable, with a reference of 0 for women and 1 for men. Residency status was included as a categorical variable; in almost all instances, residency status could easily be determined from the article text, but in a small number of cases (n = 3 individuals), it was impossible to ascertain whether an individual was living abroad or in an endemic country at the time of the consultation detailed in the reference, and thus that individual was excluded from the analyses presented here. MFD levels were included as a categorical variable, with an individual's MFD being either "Zero", "Low" or "High" (as defined in the section above). Eosinophilia status was also included as a

categorical variable, with a reference value of 0 if the individual was not eosinophilic, and a value of 1 if the patient presented with eosinophilia (with being eosinophilic defined as having a peripheral eosinophil blood count > 0.5 x 10^9 cells or a peripheral blood eosinophil count ≥ 6% as described in the main text). The number of individuals in each category for the categorical variables is detailed in Supplementary Table 3.

Supplementary Table 3: The Distribution of Individuals Across Each of the Categories Used in the Multivariate Logistic Regression Models 1 and 2

	**Model 1**<br>MFD Levels Only<br>(n = 154 Individuals)	**Model 2**<br>MFD Levels and Eosinophilia (n = 114 Individuals)
**Factor**	**Number of Individuals**	**Number of Individuals**
Males (median age)	76 (36)	53 (35)
Females (median age)	78 (29)	61 (28)
Locals living in non-endemic country	43	31
Expatriates	73	57
Locals living in endemic country	38	26
Zero mf	90	70
Low mf	48	32
High mf	16	12
Eosinophilia	Not included	98

In addition to Models 1 and 2, we also explored a second set of models, defined here as Models 3 and 4. Although Models 3 and 4 share the same structure as Models 1 and 2, they differ in their categorisation of MFD levels. Instead of three categories (Zero, Low and High), individuals were assigned to one of four categories based on the MFD recorded. These categories were: Zero (amicrofilaraemic, no mf detectable in blood smear), Low (> 0 but < 8,000 mf/ml), High (≥ 8,000 but < 30,000 mf/ml) or Very High (≥ 30,000 mf/ml). The categorisations were chosen based upon their relationship with the risk of marked or severe adverse events (SAEs) following ivermectin treatment, with ≥ 8,000 mf/ml and ≥ 30,000 mf/ml associated, respectively, with an increased risk of marked adverse events (reversible functional impairment for several days), and permanent neurological sequelae including fatal encephalopathy [4].

Analysis of these models revealed similar results to those of the models presented in the main text, although the additional granularity granted through subdivision of the MFD categories allowed resolution of a potential dose-response relationship between MFD levels and the odds of presenting atypically. Specifically, for Model 3, individuals with high MFD levels had a significantly increased risk of atypical manifestations (OR 8.27, 95% CI 1.27–53.71, p = 0.03), and individuals with very high MFD levels had an even greater risk (OR 10.84, 95% CI 1.04–113.07, p = 0.05) (Supplementary Table 4, Model 3). Neither zero nor low MFD levels were associated with an increased risk of atypical manifestations.

Similar results were observed for Model 4 (including eosinophilia status), although the significance associated with high MFD level was lost (Supplementary Table 4, Model 4), a feature likely attributable to the substantial drop in sample size that accompanied further conditioning an individual's inclusion if their eosinophilia status was reported. However, individuals with very high MFD level were still at a (marginally) significantly higher risk of atypical manifestations (OR 12.72, 95% CI 0.97-167.25, p = 0.05). The stratification of MFD into a larger number of levels led to significant reductions in the average size of each group, particularly for the high and very high categories (Supplementary Table 4), leading to substantial uncertainty in these estimates. Nevertheless, these results support the possibility of a dose-response relationship between atypical presentation and the intensity of microfilarial infection an individual harbours. The number of individuals in each category for the categorical variables considered in the analyses is provided in Supplementary Table 4 and the results of the models in Supplementary Table 5.

Supplementary Table 4: Number of individuals in each category for Models 3 and 4.

Factor	**Model 3** MFD Levels Only (n = 154 Individuals) Number of Individuals	**Model 4** MFD Levels and Eosinophilia (n = 114 Individuals) Number of Individuals
Males	76 (36)	53 (35)
Females	78 (29)	61 (28)
Locals living in non-endemic country	43	31
Expatriates	73	57
Locals living in endemic country	38	26
Zero mf	90	70
Low mf	48	32
High mf	9	6
Very High mf	7	6
Eosinophilia	Not included	98

Supplementary Table 5: Results from Multivariate Logistic Regression Models 3 and 4. Individuals are categorised into one of four categories based on their MFD levels; either Zero (amicrofilaraemic, no mf detected in blood smear), Low (> 0 but < 8,000 mf/ml blood), High (≥ 8,000 but < 30,000 mf/ml blood) or Very High (≥ 30,000 mf/ml blood).

	**Model 1** MFD Levels Only (n = 154 Individuals)		**Model 2** MFD Levels and Eosinophilia (n = 114 Individuals)	
**Factor**	**OR (95% CI)**	**p-value**	**OR (95% CI)**	**p-value**
Age	1.02 (0.99–1.05)	0.16	1.03 (0.99–1.07)	0.10
Sex	1.94 (0.89–4.20)	0.10	2.97 (1.09–8.07)	0.03*
**Demographic**				
Local living in non-endemic country	Ref	NA	Ref	NA
Expatriate	1.93 (0.73–5.09)	0.18	2.50 (0.74–8.39)	0.14
Local living in endemic country	9.17 (3.07-27.37)	<0.001*	22.62 (4.91-104.12)	<0.001*
**MF Densities**				
Zero mf	Ref	NA	Ref	NA
Low mf	1.46 (0.63–3.34)	0.38	1.62 (0.56–4.69)	0.37
High mf	8.27 (1.27–53.71)	0.03*	13.21 (0.81–215.05)	0.07
Very high mf	10.84 (1.04-113.07)	0.05*	12.72 (0.97-167.25)	0.05*
Eosinophilia	Not included	NA	0.47 (0.12–1.86)	0.28

4.3 Confidence Interval Calculation: Non-Parametric Bootstrap

In order to calculate the 95% confidence intervals (CIs) for the mean MFD of each residency status group shown in Figure 4 of the main text), an ordinary non-parametric re-sampling technique [5] was used. This process involved the following steps:

1. For each residency status group of size *n*, resampling (at random) with replacement of *n* patient MFD was conducted.
2. The mean of this newly generated sample was calculated.
3. Steps 1 and 2 were repeated 10,000 times to yield an empirical sampling distribution of the mean MFD for each different residency status group.

4. The lower and upper 95% CIs were calculated as the 2.5% and 97.5% percentiles of this sampling distribution.

Supplementary References:

[1] Valent P, Klion AD, Horny HP, *et al.* Contemporary consensus proposal on criteria and classification of eosinophilic disorders and related syndromes. J Allergy Clin Immunol, **2012;** 130(3): 607-612.e9.

[2] O'Connell EM, Nutman TB. Eosinophilia in infectious diseases. Immunol Allergy Clin North Am. **2015;** 35(3): 493–522

[3] Nana-Djeunga HC, Fossuo-Thotchum F, Pion SD, *et al*. *Loa loa* microfilariae in skin snips: consequences for onchocerciasis monitoring and evaluation in *L. loa* endemic areas. Clin Infect Dis. **2019;** pii: ciz172. doi: 10.1093/cid/ciz172 [Epub ahead of print].

[4] Gardon J, Gardon-Wendel N, Demanga-Ngangue, *et al.* Serious reactions after mass treatment of onchocerciasis with ivermectin in an area endemic for *Loa loa* infection. Lancet. **1997;** 350(9070): 18-22.

[5] Davison AC, Hinkley DV. Bootstrap Methods and their Application. Cambridge: Cambridge University Press, **1997**.
